# An overview on nanoparticle-based strategies to fight viral infections with a focus on COVID-19

**DOI:** 10.1186/s12951-022-01625-0

**Published:** 2022-10-08

**Authors:** Saman Yasamineh, Hesam Ghafouri Kalajahi, Pooneh Yasamineh, Yalda Yazdani, Omid Gholizadeh, Raheleh Tabatabaie, Hamed Afkhami, Fatemeh Davodabadi, Alireza Khanalipour farkhad, Daryoush Pahlevan, Akram Firouzi-Amandi, Kazem Nejati-Koshki, Mehdi Dadashpour

**Affiliations:** 1 Young Researchers and Elite Club, Tabriz Branch, Islamic Azad University, Tabriz, Iran; 2 Department of Medical Biotechnology, Institute of Higher Education Rab-Rashid, Tabriz, Iran; 3 Department of Biotechnology, Institute of Science, Uskudar University, Istanbul, Turkey; 4 Immunology Research Center, Tabriz University of Medical Sciences, Tabriz, Iran; 5 Department of Virology, Faculty of Medical Sciences, Tabriz University of Medical Sciences, Tabriz, Iran; 6 Department of Medical Immunology, Faculty of Medical Sciences, Hamadan University, Hamadan, Iran; 7 Department of Medical Microbiology, Faculty of Medicine, Shahed University of Medical Science, Tehran, Iran; 8 Department of Biology, Faculty of Basic Science, Payame Noor University, Tehran, Iran; 9 Determinants of Health Research Center, Semnan University of Medical Sciences, Semnan, Iran; 10 Department of Immunology, Faculty of Medicine, Tabriz University of Medical Sciences, Tabriz, Iran; 11 Pharmaceutical Sciences Research Center, Ardabil University of Medical Sciences, Ardabil, Iran; 12 Cancer Research Center, Semnan University of Medical Sciences, Semnan, Iran; 13 Department of Medical Biotechnology, Faculty of Medicine, Semnan University of Medical Sciences, Semnan, Iran

**Keywords:** SARS-CoV-2, Nanoparticles, Delivery systems, Viral infection, Nanovaccines

## Abstract

Severe acute respiratory syndrome coronavirus 2 (SARS-CoV-2) led to COVID-19 and has become a pandemic worldwide with mortality of millions. Nanotechnology can be used to deliver antiviral medicines or other types of viral reproduction-inhibiting medications. At various steps of viral infection, nanotechnology could suggest practical solutions for usage in the fight against viral infection. Nanotechnology-based approaches can help in the fight against SARS-CoV-2 infection. Nanoparticles can play an essential role in progressing SARS-CoV-2 treatment and vaccine production in efficacy and safety. Nanocarriers have increased the speed of vaccine development and the efficiency of vaccines. As a result, the increased investigation into nanoparticles as nano-delivery systems and nanotherapeutics in viral infection, and the development of new and effective methods are essential for inhibiting SARS-CoV-2 infection. In this article, we compare the attributes of several nanoparticles and evaluate their capability to create novel vaccines and treatment methods against different types of viral diseases, especially the SARS-CoV-2 disease.

**Graphical Abstract:**

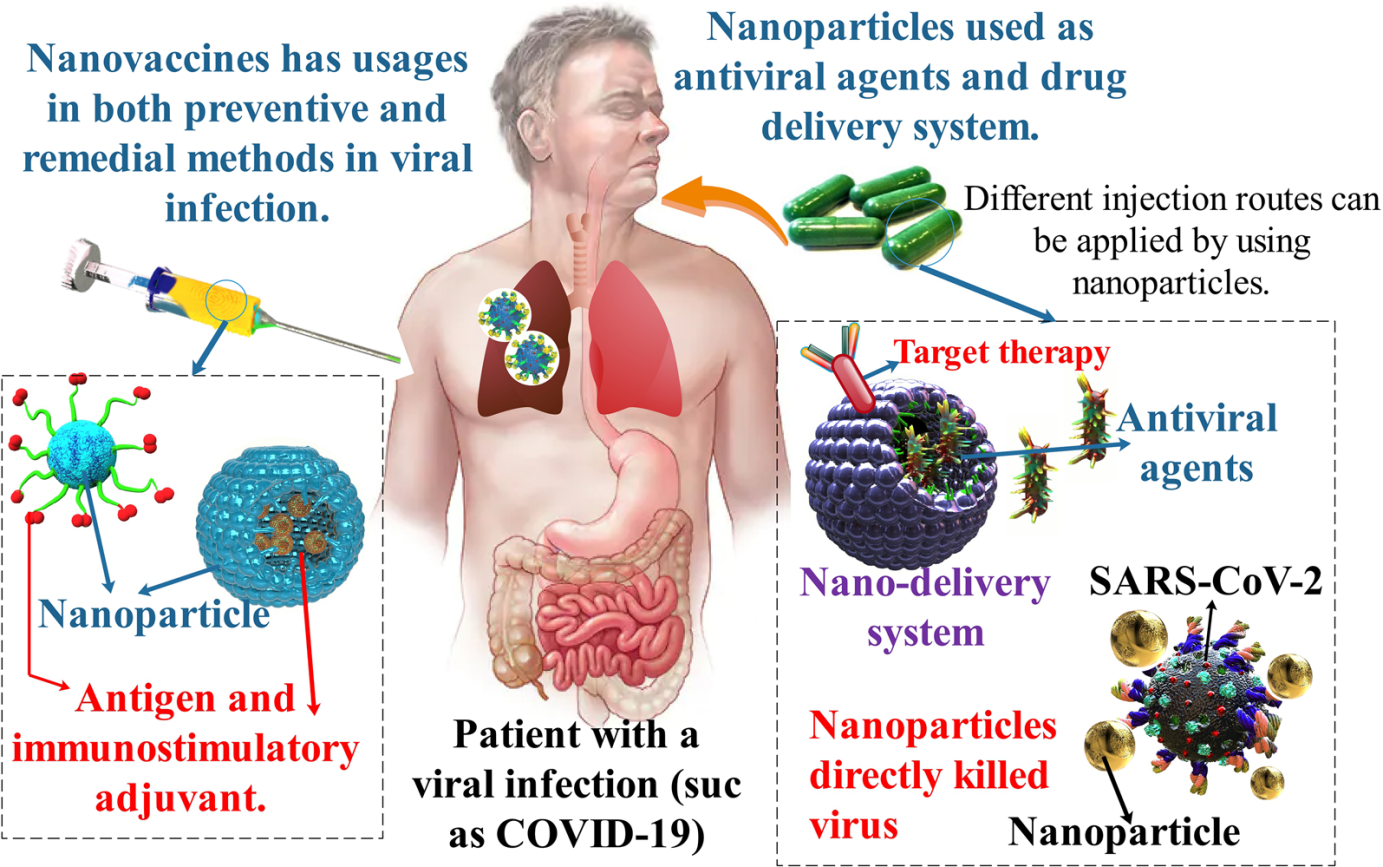

## Introduction

Severe acute respiratory syndrome coronavirus 2 (SARS-CoV-2) is the virus that causes coronavirus disease 2019 (COVID-19), a worldwide pandemic of COVID-19 resulting in over 5.8 million deaths and over 414 million infected people recovering [1, 2]. Major COVID-19 infected patients have reportedly had mild to acute respiratory infections with symptoms such as fever, cough, and dyspnea, which might emerge 2–14 days afterward exposure to the infection [3, 4]. Public-health and non-pharmaceutical interventions have been important in reducing the speed of the prevalence of the COVID-19 infection. These interventions have been important in reducing the prevalence of the COVID-19, but given their considerable societal, economic and political expenses, substitute long-time solutions are required []. A vaccine remains the more encouraging one [5, 6]. Nanotechnology, with the advancement and usage of nanoparticles (NPs)/nanocarriers, has been broadly used in a diversity of fields [7–9]. The very small dimensions of NPs allow effective entry into living organizations. Additionally, nano biomedical knowledge has been the purpose of a considerable rate of consideration, such as efficient and targeted delivery of medicines, genes, and therapeutic molecules to particular organs or cells, imaging, and accurate diagnosis of viruses at initial steps [10, 11]. The NPs of silver, gold, silver sulfide, titanium oxide, zirconium, grapheme, and polymeric compositions can be utilized as a delivery system for vaccines, which have an extraordinary ability as compared to common antigen-based vaccines [12]. Furthermore, NPs have an essential function in antiviral treatment via increasing the transfer of hydrophobic medications and increasing medicine utilization effectiveness[13]. NP-based medicines can prevent viral diseases by inhibiting virus binding and entry into the cell, suppressing viral replication, and directly deactivating viruses. Different metal NPs, polylactic acid, etc., are broadly utilized for the therapy of COVID-19 [14].

In this paper, we discussed nanostructure, which is useful in the delivery and treatment of viral infection. In addition, this study focuses on several main features of SARS-CoV-2, including epidemiology, molecular structure, viral life cycle and immune characteristic of SARS-CoV-2, vaccine/treatment method, the role of NPs in improving prevention, and therapeutic strategies of COVID-19.

## A brief overview of the important features of SARS-CoV-2 (epidemiology, molecular structure, and viral life cycle)

**Fig. 1:**
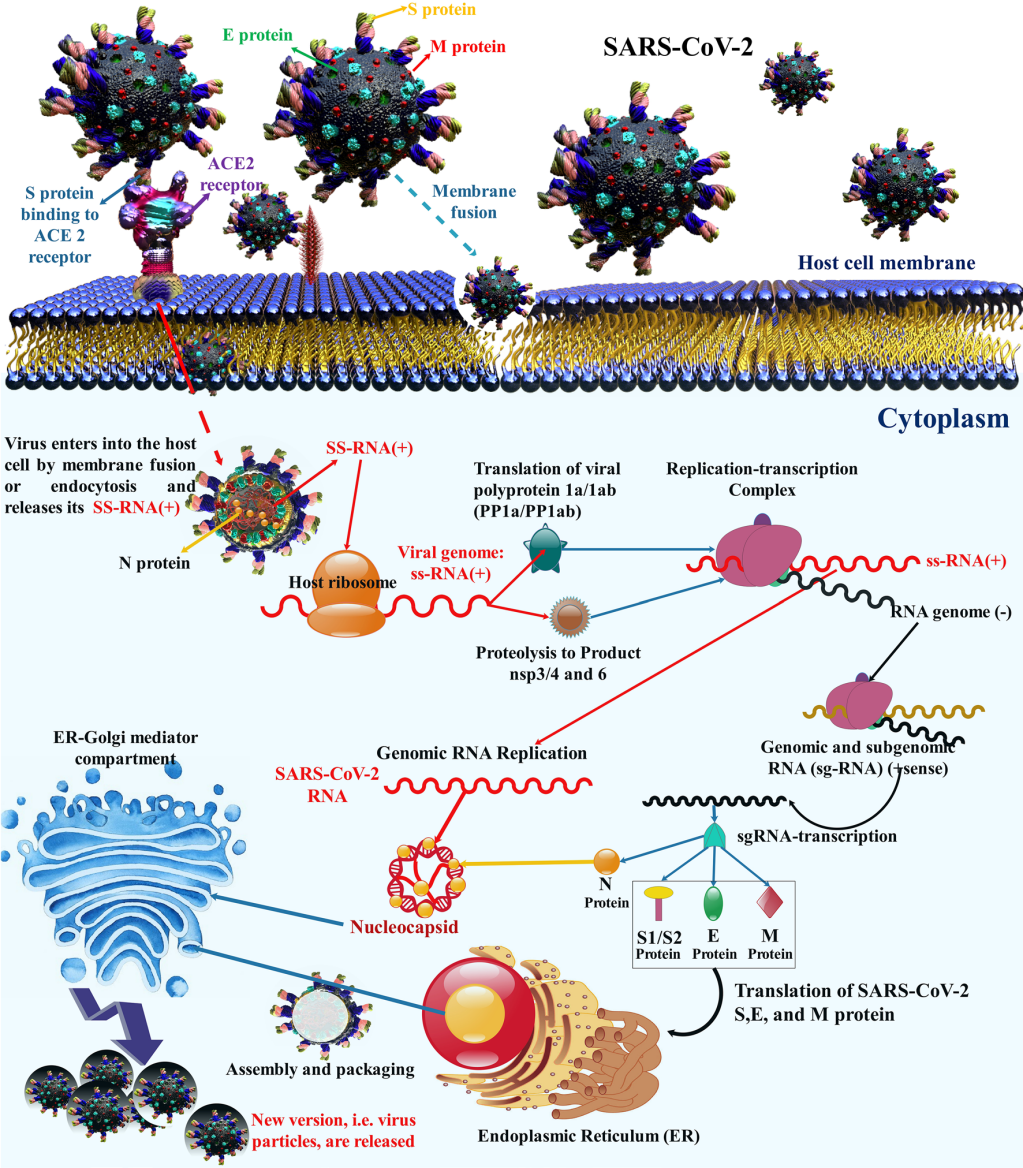
3D illustration of the structural and nonstructural protein of SARS-CoV-2 and target cell receptor (ACE2). In addition, this graphic demonstrates the entrance and replication (life cycle) of SARS-CoV-2 in target cells

Coronaviruses (CoVs) are more divided phylogenetically into 4-sort, Alpha-, Beta-, Gamma-, and Delta-CoV, and also human CoVs can be mostly separated into types, α and β-CoV [15–17]. SARS-CoV-2 belongs to β-CoVs [18, 19] and leads to the COVID-19 pandemic, which contains asymptomatic upper and lower respiratory tract diseases [20, 21]. In addition, there is powerful proof that COVID-19 in brain can lead to multiple neurological disorders and changes ranging from nonspecific to moderate to acute situations [22]. CoVs are positive-sense, single-stranded RNA (+ SS-RNA) [23]. The genome of this virus encodes several smaller open reading frames (ORFs). Structural proteins, including the spike (S) glycoprotein, envelope (E), membrane (M), nucleocapsid (N) proteins, and nonstructural proteins (NSP) are encoded by ORF [24]. The replicase gene of SARS-CoV-2 encodes two overlapping polyproteins that are necessary for viral reproduction and transcription [25]. In the 5′-UTR part, approximately more than two-thirds of the RNA comprises ORF1a/b [26, 27]. The RdRp actions in a holo-RdRp produce the whole viral genome [23]. In addition, CoVs are the main protease due to their necessary function in processing polyproteins [28]. Triggering of S needs cleavage of S1/S2 through furin-like protease and undergoes a structural alteration from prefusion to postfusion. As soon as triggered, S pursues a classic pathway between class I fusion proteins: it undergoes considerable conformational rearrangements. including shedding its S1 subunit and incorporating the fusion peptide (FP) in the host cell membrane [29]. The S2 subunit is membrane-anchored and harbors the fusion system [30]. SARS-CoV-2 S protein bind to the Angiotensin-converting enzyme 2 (ACE2) host cell [31]. As soon as prosperous entrance, the genomic RNA (sgRNA) SARS-CoV-2 acts as a transcript and lets the cap-affiliate translation of ORF1a generating polyprotein pp1a [32]. Then, the structural proteins are incorporated into membranes of the endoplasmic reticulum and transported to the endoplasmic reticulum–Golgi intermediate compartment (ERGIC). The encapsidated genome buds in the ERGIC create virions, which are afterward transported to the plasma membrane and discharged [33] (Fig. 1).

To neutralize SARS-CoV-2 infection and future prevalence, robust, repeatable, affordable, high time-efficient vaccines, and novel medicine formulations, also preventive techniques, must be produced and approved. With these issues in mind, NPs methods have been widely reported and encouraged globally as an approach to fight and inhibit COVID-19. Therefore, the SARS-CoV-2 infection needs a serious evaluation of available nanotechnologies. Also, nanomedicine methods are being utilized to produce vaccine carriers and therapy of SARS-CoV-2 [34].

## Nanoparticles-based strategies to fight against viral infections

**Fig. 2:**
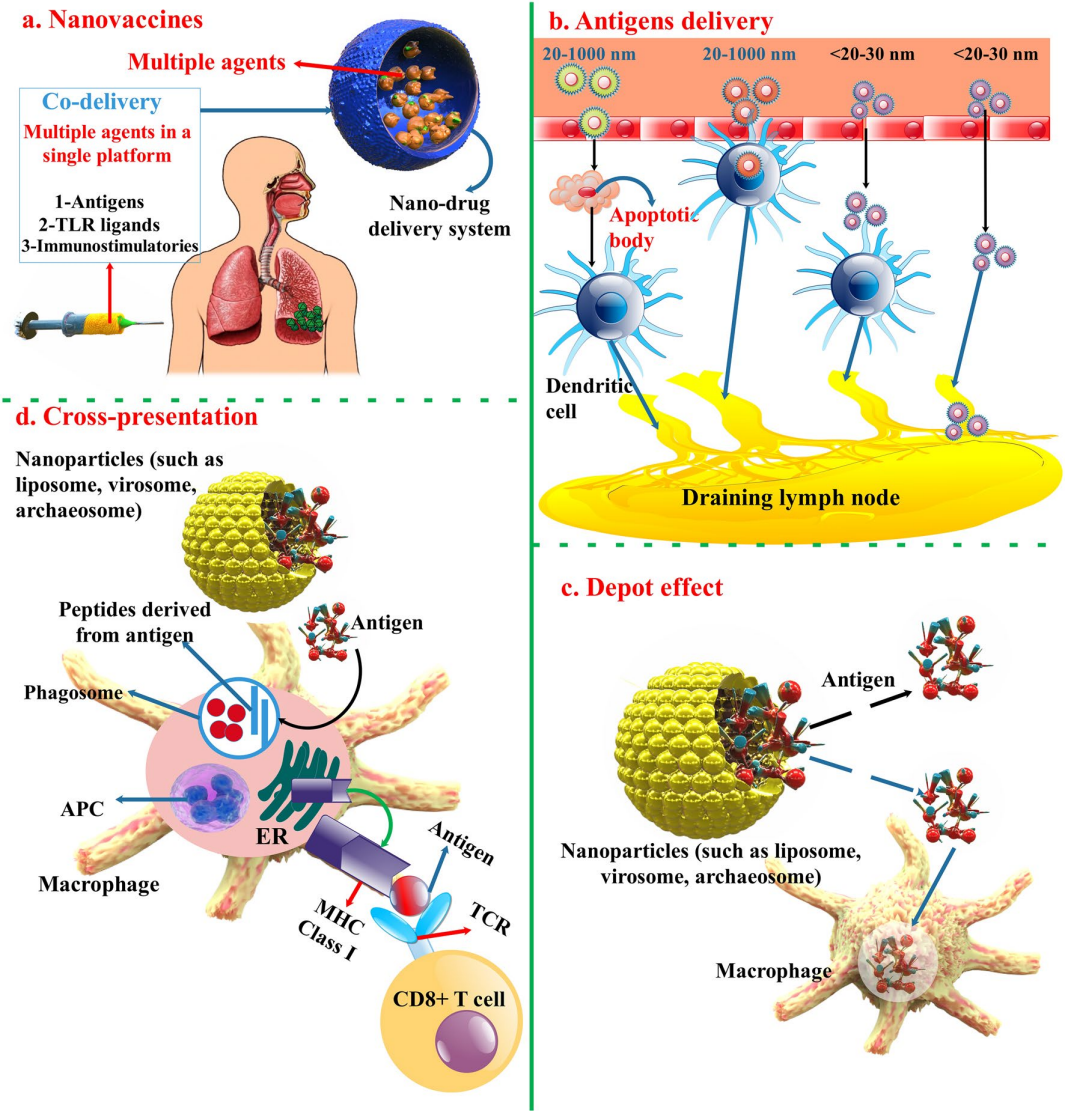
Summary of the pathways via which nanovaccines can create an immune reaction. **a** NPs can be utilized as a vaccine program for different infected illnesses because they can transport antigens and numerous immunostimulatory molecules (TLR ligands and adjuvants). The immunostimulatory action of nanovaccines is associated with different pathways, including the depot effect, gradual discharge of vaccine antigens, and absorption of antigen-offering cells. **b** Antigen transport via NPs (dimensions-related permeation and tissue or organ targeting). **c** Depot effect supplies a long-term and continuous discharge of constant antigen. **d** Cross presentation of the antigen transported via the NPs (cytosolic transport) triggers antigen particular cytotoxic T lymphocytes. Antigen-presenting cell (APC); endoplasmic reticulum (ER); T cell receptor (TCR) [38, 164]

NPs based on organic and inorganic compositions have been broadly investigated as novel vaccine methods because of their capability to induce the immune response and prepare sustained antigen discharge afterward vaccine injection. NPs can also prepare a regulated and low-speed discharge of antigens, generating a depot at the injection location supplying possible preservation versus antigen destruction [35, 36]. NP-based vaccine transfer methods designed to meet these standards have multiple benefits over conventional vaccines; (1) entrapment of antigens in NPs inhibits antigen destruction and enhance their constancy; (2) co-entrapment of antigen and immunostimulatory factor in NPs improves immunogenicity and capability of vaccines; (3) antigen-presenting cells (APCs) can easily phagocytose and procedure particles; and (4) surface decorations of NPs with functional moieties and targeting ligands allow organ- and cell-particular binding to lymphoid organs and APCs [37] (Fig. 2).

Newly, nanomedicine was developed to entrap antiviral factors to (1) enhance pharmacokinetic parameters, and bioavailability, (2) increase medicine consistency, (3) regulate/sustain medicine discharge, (4) elective transport of medicine to a specific organ, and (5) pass the blood–brain barrier [38, 39]. The functional NPs can be utilized as a wide range of antiviral factors to inhibit the primary stage of viral disease, including viral binding to host cell receptors. The second method to inhibit viruses is obstructing their permeation and entrance to target cells via altering the external membrane of the cell and protein constructions. About virus entrance into the cell, destroying their reproduction is the third efficient method to prevent the virus, which is usually attained via inhibiting the expression of some enzymes that originally assist to complete the replication of the virus genome. The last approach prevents the virus budding and excreting it from host cells [40]. In addition, NPs are recently utilized as a new strategy to directly kill the viruses by directly damaging the structure of the virus [41].

## Application of different nanoparticles against viral infections

**Fig. 3:**
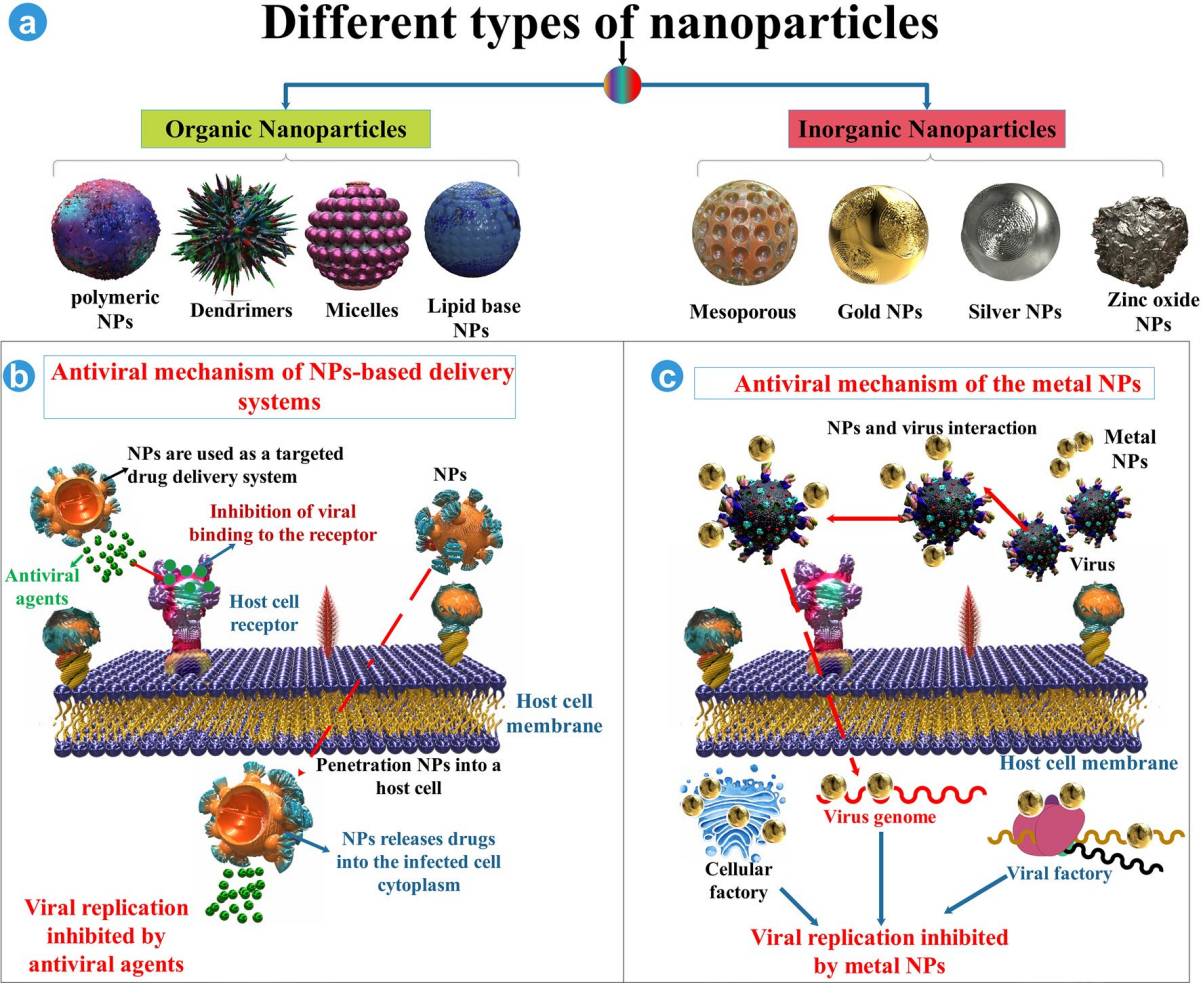
Several NPs function in treating the viral infection as antiviral factors and delivery factors. **a** Several types of inorganic and organic NPs. **b** The mechanism of the NPs as a delivery system. **c** The mechanism of the NPs as an antiviral

NPs can be classified into two classes based on the ingredients of the structure: organic and inorganic [42, 43]. NPs as drug delivery systems, including antivirals, can suppress viral reproduction in host cells via discharged antivirals from NPs obstructing target cell receptors, and released antivirals from absorbed NPs in a target cell inhibit main viral replication stages containing transcription, replication of phage DNA and synthesis of protein, and assembly [44]. Potential mechanisms include neutralization of the virus per se or indirectly, inhibition of binding of viruses to target cells, and inhibiting viral reproduction; however, they relate to the shape and kind of NPs utilized [45] (Fig. 3).

### Organic nanoparticles

Purely organic NPs have several benefits over other available NPs methods, such as self-assembly of antigens and adjuvants in physiologically mild conditions, and chemical variety for adaptable a diversity of manners, combinations, dimensions, forms, and surface functionalization. This part will investigate novel advances in organic NPs vaccine transfer methods, such as polymeric NPs, liposomes, micelles, dendrimers, solid lipid NPs, and virus-like particles (VLPs) (Table 1).

**Table 1:** The function of several organic and inorganic nanoparticles in different viral infections

Type of the viral infection	Type of the nanoparticle	Description	Refs.
HSV-1	SLNs	These NPs used as a delivery systems for encapsulation of acyclovir. One dose acyclovir SLNs have demonstrated comparative effectiveness to the several-dose regimen of ordinary acyclovir	[69]
HIV-1	Vaults	Human vaults are barrel-formed NPs by external crusts organized of 78 copies of the MVP, which line up non-covalently C- to N-terminus to supply the general vault formation	[71]
EBOV	LNP- siVP35-3	LNP-delivered siRNA as a counteraction versus this greatly deathlike human infection	[55]
Influenza	TMC NPs	The isolated HA2 and NP recombinant proteins were encapsulated in TMC NPs to effectively inhibit this infection	[72]
HSV	Micelles	Soluplus micelles remarkably increased acyclovir solvability and tolerated dilution consistency assessments. These NPs significantly reduced the penetration delay time via the cornea	[60]
IVA	THCPSi	THCPSi leads to an increase in the SaliPhe solubility, a decrease the cytotoxicity, and a considerable decrease in viral infection	[92]
H1N1	PS-GAMP	PS-GAMP powerfully increased H1N1 vaccine-triggered humoral and cytotoxic T lymphocyte immune reactions in mice via mimicking the primary stage of disease without simultaneous extra inflammation	[56]
HCV	AuNPs	This delivery system with long-acting created a more successful and sensitive therapy method for viral infection	[80]
IAV	Multivalent peptide–polymer NPs	These NPs developed to suppressing the connection of the virus to the host cell glycocalyx	[165]
H1N1	PEGylated ZnO-NPs	Exposure of these NPs with virus and bare ZnO-NPs at the greatest non-toxic densities could be caused a decrease in virus titer	[166]
H3N2	AgNPs	The inhalation route used to inject AgNPs increases survival in H3N2-infected mice	[87]
H1N1	SeNPs	SeNPs inhibit lung damage in H1N1 infected mice and repress interactivity among virus and target cell	[94]
H1N1	IO-NPs	These NPs prevent the virus from connecting to target cells in vitro	[95]
KSHV/EBV	AgNPs	These NPs triggered greater cytotoxicity in KSHV/EBV-latently diseased cells via reactivating viral lytic reproduction, which is dependent on the generation of reactive ROS production and autophagy	[88]
HBV	Ferritin NP–preS1	This NP based preS1 vaccine shows an effective antibody reaction that is both inhibitory and remedial in HBV-infected mice	[167]
HSV-2	ZOTEN	This NPs capability to entrap the virus and improve the host immune reaction against infection, and subsequently inhibit reinfection	[93]
HSV	GAunps	This NPs inhibit virus binding and infusion in the Vero cells	[81]

#### Polymeric nanoparticles

Polymeric NPs contain greatly biocompatible polymers, such as poly (lactic-co-glycolic acid) (T-lymphocyte epitopes), polyglycolic acid (PGA), and polylactic acid (PLA). Via altering the combination of the copolymer in the polymeric NPs production procedure, these NPs can act as a depot in physiological situations for sustained discharge and presentation of antigen to APCs, which is necessary for mucosal injection [46, 47]. Ivermectin (IVM) medicine was entrapped in PLGA-b-PEG polymers NPs to inhibit of transmission of the Zika virus (ZIKV). The core–shell construction of these NPs lets them encapsulate and transport weakly water-soluble medicines, including IVM, leading to prolonged circulation half-life for the medication, discharge medicines at a sustained amount, and functionalization by targeting ligands to regulate the delivery system to target particular zones. This nanomedicine is administered through the oral route [48]. NPs can also directly interfere with and suppress viral replication via the multivalent presentation of small molecules that prevent viral assembly processes while selectively eliminating latently HIV-infected resting memory CD4 + T cells. [49]. Subsequently, T-lymphocytes-membrane-covered NPs (TNPs) inherit T lymphocytes surface antigens important for HIV targeting [50]. In other investigations, that incorporated the plasma membranes of uninfected CD4 + the resulting TNP mimicked the parent CD4 + T lymphocytes onto PLGA cores and T cells. This method induced autophagy in HIV-infected cells and decreased cell-related HIV-1 [49]. Multivalent peptide–polymer NPs, which is a dendritic polyglycerol scaffolds and excellent suited for a multivalent exposure, connecting with influenza A virus (IAV) via virus surface hemagglutinin to suppress attachment of the IAV to the target cell. In other investigations, investigators developed new polymeric NPs, densely combined with different ligands to selectively attach to ACE2, as advanced nanovectors for targeted medicine transfer such as remdesivir, in SARS-CoV-2 infection. Remdesivir-encapsulated in targeted NP (TNP) exhibited increased antiviral efficacy versus COVID-19. In addition, empty TNP showed an essential antiviral function, possibly owing to a direct competitive mechanism with viral particles for the ACE2 connection location [51]. Fluoxetine hydrochloride (FH), an antidepressant medication, can inhibit SARS-CoV-2 infection. FH was encapsulated in lipid polymer hybrid NPs (LPH) to increase its effectiveness in the treatment of the SARS-CoV-2 infection [52].

#### Liposomes

Liposomes are spherical nanocarriers containing one or multiple lipid bilayers prepared via hydrophilic and hydrophobic interplays with the aqueous phase. Two significant benefits of liposomes, in medicine transfer of living organisms, are biocompatibility and biodegradability, which are owing to lipid features [53, 54]. For example, Lipid NPs (LNP) have been used in the delivery of siVP35-3 for rhesus monkeys infected with the Ebola virus (EBOV), and these NPs increased the targeted therapy and stability of siRNA in this considerably fatal human infection [55]. Wang, et al. produced pulmonary surfactant (PS)-biomimetic liposomes entrapping 2′,3′-cyclic guanosine monophosphate-adenosine monophosphate (cGAMP), an agonist of the IFN gene stimulus STING. The adjuvant (PS-GAMP) strongly completed influenza vaccine-elicited humoral and cytotoxic T lymphocyte immune reaction in mice via mimicking the primary stage of viral diseases lacking simultaneous surplus inflammation. Two days afterward, inhalation injection by PS-GAMP-adjuvanted H1N1 vaccine, powerful cross-preservation was induced versus H1N1 viruses for at minimum 6 months, whereas protecting lung-inhabitant memory and cytotoxic T lymphocytes [56]. In other investigations, developed RBD-encoding mRNA (RBD-mRNA) encapsulated in liposomes (LPX/RBD-mRNA). This method can express RBD in vivo and effectively elicit SARS-CoV-2 RBD particular antibodies in the injected mouse model, which effectively inhibits COVID-19 [57]. Researchers are developing the COVID-19 vaccine with three different lipophilic adjuvants encapsulated in liposomes. The results showed that MPLA-adjuvanted liposome NPs vaccines whole elicited a strong particular antibody reaction against SARS-CoV-2 infection [58].

#### Micelles

Micelles are spherical NPs delivery systems combined with a surfactant monolayer, and their dimensions are the limited area between 10 and 1000 nm. Polymeric micelles (PMs) are colloidal delivery methods prepared via the molecular gathering of block copolymers with amphiphilic properties in a watery medium. PMs are known for their excellent medicine-loading capability and exclusive disposition features in the body. The determined chemistry of the block copolymers leads to the chemical combination of several medicines with polymeric chains [59]. For example, soluplus or solutol polymeric micelles have been used to enhance acyclovir solubility, corneal penetrance, and sclera permeation of drugs for cornea and sclera tackling with herpes simplex virus (HSV). Solutol micelles enhanced their size when combined with drugs. In this method, quantities of medication penetrated via the sclera were approximately 10 times higher than free drug, which opens the probability of medicine transfer to the posterior eye section [60]. The receptor connection and proteolysis of the S protein of COVID-19 discharge its S2 subunit to rearrange and catalyze viral-cell fusion. SARS-CoV-2 S proteins fusion peptide alters from inherent rearrange in solution into a wedge-formed conformation incorporated in bilayered micelles, based on chemical changes [61]. S protein comprises a single-span transmembrane (TM) domain and is important for viral infection. This TM domain was reconstructed in detergent micelles. Though this type of micelles may not be a perfect method for constructional and functional investigations of membrane proteins, used as a beneficial membrane method to comprehend the second construction of a membrane protein [62].

#### Dendrimers

Dendrimers are highly branched structures containing dendron monomers. These symmetrical macromolecules have a limited dimension of 10 to 100 nm. Dendrimers have diverse functional groups on their surfaces, and they inhibit virus penetration to cells by their interaction with viral particles and cell protection via their different functional groups [63, 64]. Three kinds of polycationic dendrimers comprising primary amine were utilized to evaluate their antiviral function with the MERS‐CoV (Middle East respiratory syndrome coronavirus) plaque suppression test. The hydroxyl polyanionic group demonstrated a 17.36% to 29.75% reduction in MERS‐CoV plaque forming. The most effective suppression of MERS‐CoV plaque-forming was observed via G (1.5)‐16COONa (40.5% repression), followed via G(5)‐128SA (39.77% repression). Polyanionic dendrimers can be added to antiviral provisions to increase the transport of antivirals, and also the inherent antiviral action [64]. DanielSepúlveda-Crespo, et al. developed a cell-based method to screen a battery of polyanionic carbosilane dendrimers (PCDs) to recognize complexes with antiviral activity versus HCV and display that they prevent efficient virus infusion of main HCV genotypes. Remarkably, one of the PCDs permanently destroyed infectious virions [65]. In other investigations, produced a treatment method for SARS-CoV-2 infection by using an improved anti‐COVID-19 siRNAs encapsulated in a new safe peptide dendrimer KK‐46 as a delivery system. The result showed that topical therapy via intranasal injection of the improved siRNA‐peptide dendrimer formulation can decrease viral reproduction and improve COVID-19‐induced lung inflammation [66]. Orpheris company is evaluating a remedial including N-acetyl-cysteine connected to the inactive dendrimer OP-101 in acute SARS-CoV-2 infected patients. In a stage II clinical trial (NCT04458298), this treatment method was shown to decrease SARS-CoV-2-dependent inflammatory cytokine storms [67].

#### Solid lipid nanoparticles

Solid lipid NPs (SLNs), are solid core lipid nanocarriers, which can encapsulate both hydrophilic and hydrophobic medicines. The unique property of SLNs is that they can deliver a diversity of treatment agents such as small medicine molecules, big biomacromolecules, genetic substantial, and vaccine antigens [68]. SLNs have been used in acyclovir delivery to treat HSV. As a result, this investigation displayed that the developed one dosage acyclovir SLNs have shown comparative efficiency to the multiple-dosage regimen of conservative acyclovir. And also, these NPs have the capability carriers for oral treatment in the therapy of HSV-1 infection [69]. Researchers produced a novel aerosolized SLNs-formulation of favipiravir (FPV) as an antiviral factor versus SARS-CoV-2. The results showed that FPV-SLNs were suitable for intranasal injection [70].

#### Other organic nanoparticles

“Vaults” are everywhere expressed endogenous ribonucleoprotein NPs by possible usefulness to selective medicine delivery. Human vaults are barrel-formed NPs with outside layers organized of 78 versions of the major vault protein (MVP), which equal non-covalently C- to N-end to prepare the general vault construction. Fulcher et al. used recombinant human vault NPs to target the delivery of antiviral medicines, including zidovudine, tenofovir, and elvitegravir, which are without an intermediary connection to vaults, in HIV-1. This drug delivery system is useful for effectively targeting the human peripheral blood mononuclear cells (PBMC), mainly DCs, monocytes/macrophages, and triggered T lymphocytes [71]. Rungrojcharoenkit et al. produced influenza NP structures utilizing trimethyl chitosan NPs (TMC NPs) as the delivery system of recombinant influenza hemagglutinin subunit 2 (HA2) and nucleoprotein. The isolated HA2 and recombinant nucleoproteins were entrapped in TMC NPs to create HA2-TMC NPS and nucleoprotein-TMC NPs, in order. TMC NPs encapsulated with influenza subunit antigens or all deactivated influenza virus enhanced immune reactions and the efficacy of inhalation route injected vaccines in the mice. HA2-TMC NPs, nucleoprotein-TMC NPs, and HA2-nucleoprotein-TMC NPs (influenza NPs structures) displayed no toxicity in human intranasal epithelium cells (HNEpCs) [72]. Researchers are designing phage capsid NPs as hard scaffolds which are functionalized via a conformationally determined offering of the sialic acid (Sia) ligands to couple the connection location of the trimeric hyaluronic acid (HA). These capsid NPs coat the whole IAV envelope, inhibiting its targeting of the target cell as imagined via cryo-electron tomography [73]. Nanocellulose/polyvinyl alcohol/curcumin (CNC/PVA/curcumin) NPs were produced as a nanotechnology treatment method with increased medicine loading for intranasal injection of antiviral agents SARS-CoV-2 infection. The results showed that the increased loading of curcumin in nanocellulose will supply an encouraging NPs-based solution for the therapy of SARS-CoV-2 infection [74]. In other studies, nano entrapped polyphenolic compounds were developed as a therapeutic agent against COVID-19. These compounds were entrapped in moieties of bovine serum albumin (BSA) and next were covered via chitosan as a mucoadhesion polymer. NPs created with BSA have features such as non-toxicity, well consistency, great medicine capacity, and potential to entrap hydrophobic and hydrophilic medicines [75].

### Inorganic nanoparticles

The majority of inorganic NPs have a smaller dimension, enhanced constancy, regulated adjustable, increased penetrance, excellent medicine loadings, and an activated discharge profile, perfect for antigen transport as a vaccine. These novel productions are usually produced with an inorganic core and an organic outside covering to provide hybrid inorganic NPs [76] (Table 1).

#### Gold nanoparticles

Gold NPs (AuNPs) can have a main function in the vaccine field as adjuvants, decreasing toxicity, increasing immunogenic action, and developing consistency of vaccine in storing, and have a high ability as delivery systems for the creation of a high variety of completely synthetic vaccines [77, 78]. When preparing AuNPs, methods, dimensions, and form have an essential effect on antigen exposure, cellular absorption, blood clearance, bio-distribution, and immunological reaction [79]. IFN-alpha delivery, along with AuNPs and HA, has been used in the therapy of HCV. HA-AuNP/IFN-α compound considerably increased the expression of 20, 50-oligoadenylate synthetase 1 (OAS1) for innate immune reactions against viral disease in the liver tissue [80]. Halder et al. developed quasi-spherical AuNPs by utilizing ultrasound-induced fast decrease in gallic acid (GA), resulting in greatly monodispersed AuNPs (GAuNPs) for inhibited HSV infections in Vero cells. GAuNPs inhibited viral binding and fusions in the Vero cells. Nontoxic and biocompatible AuNPs were offered as a harmless alternative in viral chemotherapy [81]. In other investigations, researchers developed a particular S protein of the SARS-CoV-2 epitopes conjugated with AuNPs. The results showed that subcutaneous injection of this nanovaccine increased the humoral response [82]. Chen, et al. suggested a vaccine that binds the immunomodulation of AuNPs, capped with polysaccharide that has antiviral attributes, encapsulated with S or N proteins from SARS-CoV-2 [83].

#### Silver nanoparticles

Silver NPs (AgNPs) are the most efficient in conflict with pathogenic among all metallic NPs [84]. Several investigations have shown that AgNPs could simply enter living cells. The dimensions and form of AgNPs play a very significant role in antiviral action. Numerous researches have displayed that those sizes smaller than 10 nm generate much more reactive surfaces. The shape can also differ—for instance, triangular, bar, or spiral—which strongly influences the mechanism of the viral act; those of the sphere-shaped and cylinder-shaped kinds are more phagocytosed [85]. These NPs bind to the viral genome and as a result inhibiting the action and interaction of several viral and cellular agents responsible for replication leading to the suppression of viral replication and virus release [86]. In other studies, Madin-Darby canine kidney cells infected by AgNP-remedied H3N2 influenza virus displayed higher survival and no apparent cytopathic effects contrasted with an influenza virus healthy group and a group remedied by the solvent utilized for the provision of the AgNPs. These NPs remarkably suppressed the H3N2 replications and decreased cell apoptosis created by the H3N2 influenza virus [87]. In other research, spherical AgNPs with a size of 25 nm can inhibit Kaposi’s sarcoma-associated herpesvirus (KSHV) early infection via per se annihilating virion subunits; it as well successfully prevents colony development and relatively suppresses the growth of KSHV-related primary effusion lymphoma (PEL) tumors in xenograft mouse [88]. In other investigations, AgNPs were evaluated in vitro and demonstrated to have a preventing efficacy on COVID-19 in cultured cells. Therefore, researchers evaluate the effectiveness of mouthwash and nose rinse with ARGOVIT® AgNPs, in the inhibition of COVID-19 in health personnel. The results showed that the mouth and nasal rinse by AgNPs assist in the inhibition of COVID-19 in health workers who are exposed to patients detected with SARS-CoV-2 infection [89]. In other investigations, researchers developed green synthesized AgNPs by utilizing strawberry and ginger methanolic extracts to suppress COVID-19. The methanolic strawberry extract and the green synthesized AgNPs of ginger demonstrated the most excellent antiviral acting versus COVID-19 [90].

#### Other inorganic nanoparticles

Mesoporous silica NPs (MSNs) are nanoporous silica globes 100–200 nm in diameter with holes filled with natural prodrugs, functionalized by amino groups, and filled by natural compounds of shikimic acid (SH), quercetin (QR) (The MSNs-NH2-SH and MSNs-NH2-SH-QR2) or together, which showed a powerful antiviral reaction against H5N1 infection. These NPs repressed cytokines and nitric oxide generation through about 50% for MSNs-NH2-SH-QR2 (comprising both SH and QR) [91]. Thermally hydrocarbonized porous silicon (THCPSi) has been used in saliphenylhalamide (SaliPhe) delivery to inhibit IVA in vitro. NPs drugs delivery system based on porous silicon indicated enhanced dissolution of the researched IAV inhibitor medicine SaliPhe and demonstrated great in vitro resistance, less cytotoxicity, and a significant decrease of viral load in the lack of organic solvents [92]. Agelidis et al. demonstrated that exclusively produced zinc oxide (ZnO) tetrapod NPs (ZOTEN) display a powerful microbivac effect versus HSV-2 in a murine sample of genital infection. These NPs are capable of attacking the virus subunits and influencing the host immune system, showing their new and multifunctional antiviral attributes with hopeful preventive and therapeutic efficacy [93]. Surface altered selenium NPs (SeNPs) via arbidol (Se@ARB) by better viral inhibition features than medicine resistance produced in a trial. Se@ARB affected the interplay between the H1N1 influenza virus and the target cells by repressing the acting of HA and neuraminidase (NA). In addition, this treatment method could inhibit H1N1 from transmitting the infection to MDCK cells and repress DNA destruction and chromatin condensation [94]. Iron oxide NPs (IO-NPs) are used to inhibit the pandemic influenza strain A/H1N1/Eastern India/66/PR8-H1N1. The viral inhibition function of the IO-NPs was shown to the reduced proportion of viral suspensions afterward therapy by the IO-NPs. The antiviral activity of IO-NPs displayed more excellent suppression at a lesser dose can be owing to small dimensions that simply react to the virus [95].

AuNPs, AgNPs, CuNPs, ZnNPs, and Fe2O3 NPs are efficient versus COVID-19. A potential mechanism of function of these NPs versus CoVs is a disorder of the outer surface of CoVs. The Ayurvedic Bhasma formations are innovative metal NPs. These metal NPs are nontoxic, constant in the solid phase, and have a great biological function. Ayurvedic metal NPs, could be used as new antiviral factors versus COVID-19 for their anti-inflammatory, immunomodulatory, antiviral, and adjuvant functions [96]. Other investigations, showed that different graphene-NPs (GNPs), including intact graphene (IG), defective graphene (DG), and graphene oxide (GO), suppressed SARS-CoV-2M proteins. DG and GO interfered with M protein more powerfully, leading to disabling M proteins and suppressing their expression efficiently via annihilating the active pocket of M protein [97].

## SARS-CoV-2 immunopathology (innate and adaptive immune response)

**Fig. 4:**
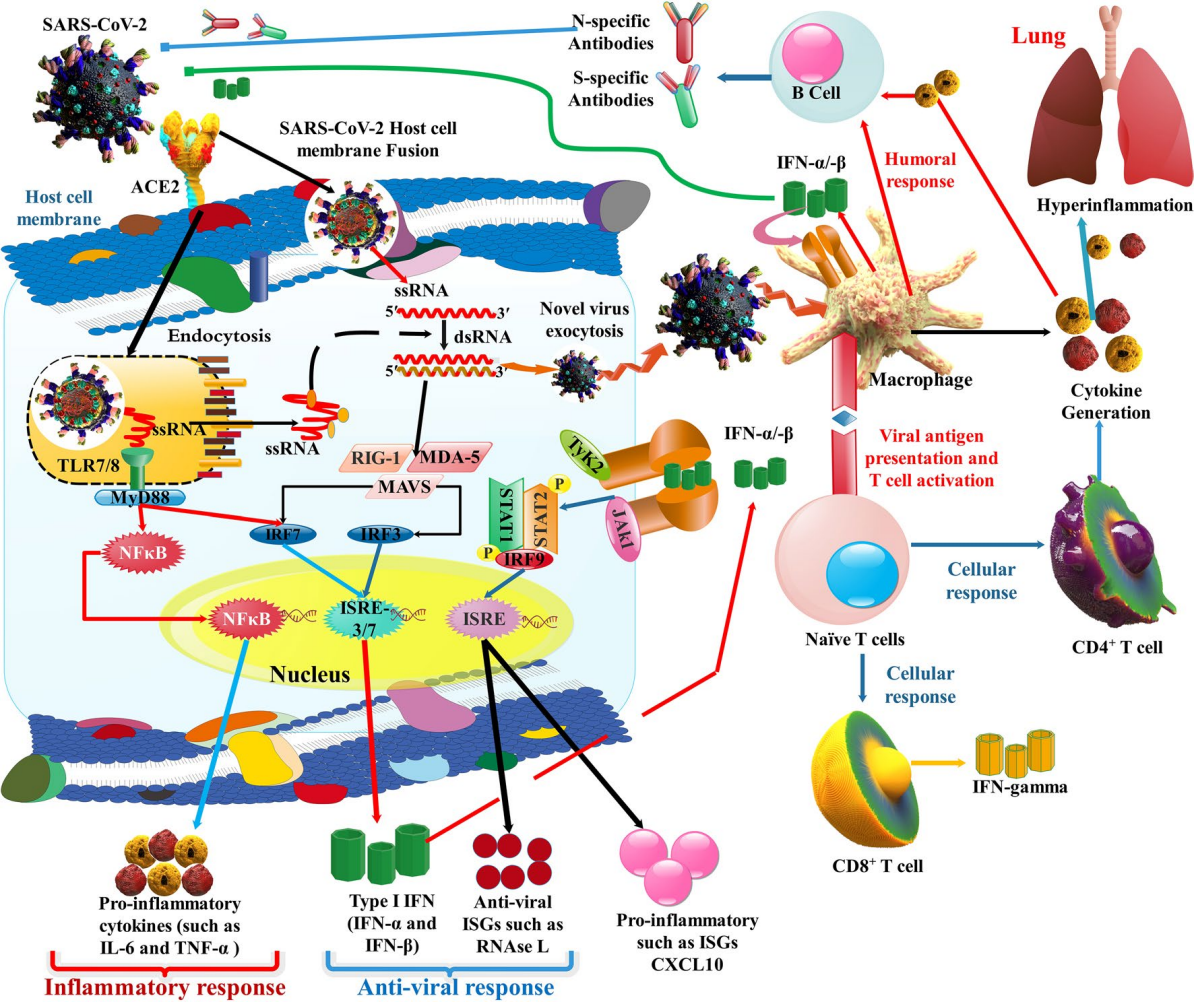
This illustration demonstrates the possible immunopathogenesis in COVID-19 infection (innate immunity, adaptive immunity, and humoral immunity)

Innate immunity commenced by detecting pathogen-associated molecular patterns (PAMPs) through host pattern recognition receptors (PRRs). The IFN-I pathway is an essential section of the innate immune reaction. PRRs diagnose many viruses, and this detection activates a downstream antiviral cascade such as microRNAs antiviral action [94, 98, 99]. The CoVs ds-RNA could be recognized via the retinoic acid-inducible gene I-like receptors (RLRs), comprising the RIG-I and, or melanoma differentiation gene 5 (MDA5) into the cytoplasm, or via TLRs within the endosome. The 2 caspase recruitment domains (CARD) of RIG-I and MDA5 could react to the adapter mitochondrial antiviral signaling protein (MAVS), which consequently induces the 2 IKK-associated kinases, TANK-binding kinase 1 (TBK1), and inducible IκB kinase (IKKi), together of which phosphorylate IFN regulatory factor 3/7 (IRF3/7). Afterward phosphorylation and dimerization, IRF3/7 replaces in the nucleus to trigger the expression of IFN-α/β. Simultaneously, MAVS induces TANK1 via TRAF6 and triggers the NF-κB signaling mechanism, which could increase cytokines generation. On the other hand, PAMPs could be recognized via TLRs, and the downstream adapter proteins TRIF or MyD88 could signal to activate cytokines and chemokines generation [100]. Signaling at these cell receptors stimulates cytosolic translocation of several nuclear transcription agents, including NF-kB and the activating protein-1 (AP-1) to the cell nucleus, the transcription of genes, and expression of critical inflammatory reaction proteins, including CRP, proinflammatory cytokines, and chemokines, also discharge of soluble agents related on the IFN protein stimulator gene (ISGs) that encodes IFNs function in virus regulator, activation anti-virus mode [101]. Afterward viral induction, IFNs are induced via the detection of PAMPs with PRRs, endosomal (TLR3 and TLR7), and cytosolic (RIG-I) receptors, such as triggering and phosphorylation of the JAK/STAT pathway and the creation of the heterotrimer compound STAT1-STAT2-IRF9 (ISGF3) that is involved in the stimulation of the genes accountable for the reactions to IFNs in the promoter section of the ISG. STAT1/2 generate a compound with IRF9, and together they transfer to the nucleus to begin the transcription of ISGs under the regulation of IFN-stimulated response element (ISRE) comprising promoters (Fig. 4) [101].

In COVID-19 infected patients, the reports have shown that primary plasma measures of IL-1β, IL-1RA, IL-7, IL-8, IL-10, IFN-ɣ, monocyte chemoattractant peptide (MCP)-1, macrophage inflammatory protein (MIP)-1A, MIP-1B, granulocyte-colony-stimulating factor (G-CSF), and TNF-α are enhanced in COVID-19 patients. Moreover, the rate of helper T lymphocytes and cytotoxic T lymphocytes inhibitor (CD3+, CD8+), and regulatory T cells (Tregs) are lower than normal rates; however, T helper and Tregs in acute patients are significantly lesser than in mild cases [102, 103]. The SARS-CoV-2 binding to the target cell by the ACE-2 and is recognized (essentially) via TLR7. TLR7 activity results in the generation of IFN-α, and the release of IL-12 and IL-6. This leads to the creation of CD8 + -particular cytotoxic T lymphocytes and, via the CD4 + lymphocytes, results in the generation of antigen-particular B lymphocytes and antibody generation (IgM, IgG, and IgA (pan-immunoglobulin (pan-Ig)). In TLR7 signaling-associated cytokine discharge, IL-6 can play a significant function. It is essential in the formation of follicular CD4 + lymphocytes, TH17 subgroup deflection, and the creation of prolonged lived plasma cells [104].

## Therapeutic and prevention procedures of the SARS-CoV-2 infection

Mutations in the SARS-CoV-2 genome make it challenging to produce treatment methods. On the other hand, the high prevalence of this virus has led to the rapid development of an effective treatment method [105]. Therefore, different medical, social, and engineering techniques have been proposed to face the SARS-CoV-2 prevalence that contains treatment, prevention, diagnosis, and prediction methods. In addition, essential therapies were presented, including antiviral medicines (e.g., dexamethasone [106], Favipiravir [107], and Remdesivir [108]), antibiotics, oxygen therapy, and antibody therapy [109, 110]. In addition, utilized convalescent plasma technique for the therapy of COVID-19, which is an immunotherapy method by viral-particular antibodies [111]. Mainly, SARS-CoV-2 infection leads to remarkable injury to the lungs; therefore, pulmonary medication transfer must be included as a curative method for important action. In this context, aerosol-based intranasal injection route designs might be an improved method which is not only patient compliant but can as well show the quick relief over a specified period. In its pure form, bilirubin has difficulties with its solubility; therefore, its injection in NP form provides improved solubility and enhanced effectiveness. Bilirubin nanomedicine (BNM) as aerosol-based medication delivery method can carry payload directly to the lungs and decrease the complications of SARS-CoV-2 leading to enhanced patient condition [112].

Based on reports of the World Health Organization (WHO), on Jan. 31, 2022, vaccine candidates were in clinical assessment to remedy COVID-19, 114 vaccines in clinical assessment, and 48 candidate vaccines have attained the final phases of the trial [5, 113]. These vaccines include inactivated vaccines, nucleic acid vaccines, vector vaccines, and subunit vaccines. Inactivated vaccines are broadly utilized to inhibit emerging infectious diseases (EID), and the partly great speed of the generation of this type of vaccine makes it a hopeful method for COVID-19 vaccine production [114, 115]. These vaccines are complete virus formations that are chemically inactivated with beta-propiolactone and formaldehyde. However, they are no longer replication-ability, virus integrity is protected, and acts as an immunogen that is S-particular, RBD-particular and N-particular. When injected, inactivated vaccines induce preservative immune reactions against the pathogen. This type of vaccine is obtained from viruses grown in culture and next chemically inactivated, which can deliver stably expressed, structurally native antigenic epitopes (such as Sinopharm and Sinovac vaccines) [116]. Nucleic acid (RNA and DNA) vaccines are facile to produce, which allows their fast progress as vaccines. The genome encoding for a specific protein can be simply formed as DNA or RNA and introduced in human cells to generate several copies of the immunostimulatory viral antigenic proteins. These antigens, firstly encoded via the nucleic acid, can induce both humoral and cell-mediated immune reactions upon expression following cellular absorption. This type of vaccine has self-adjuvating attributes and hence can generate both adaptive (antigen-based) and innate immune reactions. In contrast, most other vaccine kinds require an adjuvant to attain a similar purpose. Nucleic acid-based anti-SARS-CoV-2 vaccines may have benefits over conventional vaccines such as (1) The great power of mRNA vaccines is able of producing potent antiviral neutralizing antibodies via triggering both CD4 + and CD8 + T-cells with only one or two low-dose vaccinations; (2) Due to its destruction procedure in cells, mRNA-based vaccines decrease the danger of infection and mutations are caused by insertion [117]. Vector vaccines can be widely divided into two categories: replication-incompetent vectors and replication-incompetent vectors vaccines. Replication-incompetent vectors show a big group of vaccines in expansion. This type of vaccine is usually based on another virus that has been designed to express the S protein and has been inactivated from reproduction in vivo via the elimination of sections of its genome. Replication-competent vectors are usually derived from weakened or vaccine strains of viruses that have been prepared to express a transgene, in this case, the S protein. Since viral vector vaccines lead to endogenous antigen generation, they are more likely to stimulate both humoral and cellular immune reactions. These vaccines can be advanced and generated rapidly on a wide-ranging and do not need extremely low temperatures for transport and storing. However, pre-existing immune reactions to the vector can restrict the capability of the vector to carry genetic material to target cells and thus decrease the efficiency of the vaccine [118–120]. Subunit vaccines are vaccines produced based on synthetic peptides or recombinant proteins. Dissimilar to inactivated or live-attenuated viruses and certain viral vectored vaccines, this vaccine kind mainly comprises particular viral antigenic parts; however, without containing each ingredient of infectious viruses, removing the worries of imperfect inactivation, improve pathogenicity, or pre-existing immune reaction. Like DNA or VLP-based vaccines, subunit vaccines are usually harmless without causing possible adverse immune reactions, making them hopeful vaccine candidates. Furthermore, subunit vaccines may target particular, well-defined neutralizing epitopes with enhanced immunogenicity and, or effectiveness. For example, Novavax has advanced and generated immunogenic virus-like NPs based on recombinant expression of the S-protein, while Clover Biopharmaceuticals is producing a subunit vaccine containing a trimerized SARS-CoV-2 S-protein utilizing their patented Trimer-Tag® method, though some full-length S-proteins for SARS as well cause enhanced infection and eosinophilic penetration [5, 121] (Table 2).

**Table 2:** Specifications of some SARS-CoV-2 vaccines in the advanced clinical development phase

Vaccine platform	Vaccine information	Developer	Phase and registered trials	Approved	Number of doses	Timing of doses	Clinical Trials.gov Identifier	Refs.
Inactivated vaccine	The inactivated COVID-19 vaccine (Sinovac: CoronaVac) with aluminum hydroxide	Sinovac	III 40 trials in 10 countries	56 countries	2	0, 28 days	NCT0445659	[168]
	Placebo/Aluminum Adjuvant of inactivated COVID-19 vaccine (Sinopharm (Beijing): Covilo vaccine (known as BBIBP-CorV (Vero Cells))	Beijing Institute of Biological Products/Sinopharm	III 38 trials in 16 countries	98 countries	2	0, 21 days	NCT04560881	[169]
Non-Replicating Viral Vector	Oxford/AstraZeneca: Vaxzevria (also known as AZD1222, ChAdOx1 nCoV-19)	University of Oxford/AstraZeneca	III 70 trials in 33 countries	148 countries	2	0, 28 days	NCT04516746	[170, 171]
	Adenovirus Type 26 vector (Janssen (Johnson & Johnson): Ad26.COV2.S and also known as Ad26COVS1, JNJ-78436735)	Janssen Pharmaceutical Companies	III 25 trials in 25 countries	113 countries	2	0, 56 days	NCT04908722	[172]
Protein Subunit	Novavax: Nuvaxovid (also known as NVX-CoV2373). Full length recombinant COVID-19 virus glycoprotein NP vaccine adjuvanted with Matrix-M1	Novavax	III 20 trials in 13 countries	39 countries	2	0, 21 days	NCT04611802	[173]
	National Vaccine and Serum Institute: Recombinant SARS-CoV-2 Vaccine (CHO Cell) (also known as recombinant COVID-19 Vaccine (CHO cell, NVSI-06–08). The adjuvanted recombinant protein (RBD-Dimer) expressed in CHO cells	National Vaccine and Serum Institute	III 3 trials in 2 countries	1 countries	2–3	0, 28, 56 days or 0 + 28 days	NCT05398848	[174]
RNA based vaccines	LNP-encapsulated mRNA vaccine (Moderna: Spikevax also known as mRNA-1273, and Elasomeran vaccine)	Moderna/NIAID	III 68 trials in 24 countries	88 countries	2	0, 28 days	NCT04470427	[175]
	3 LNP-mRNAs vaccine (Pfizer-BioNTech COVID-19 BNT162b2 Vaccine or Tozinameran)	BioNTech/Fosun Pharma/Pfizer	III 90 trials in 29 countries	148 countries	2	0, 28 days	NCT04848584	[176]
Virus-like particles	Medicago: Covifenz and also known as CoVLP, MT-2766, Plant-based VLP. Plant-derived VLP adjuvanted with AS03	Medicago Inc	III 6 trials in 6 countries	1	2	0, 21 days	NCT05040789	[177]

## Nanoparticle vaccines against SARS-CoV-2

Nanovaccinology has been used in preventive and remedial methods and can be utilized for each enhancement antigen processing or offering and, or as an immunostimulatory adjuvant [122]. Main NPs platforms contain lipid base NPs (LNPs) and VLPs. LNPs comprising ionizable lipids have been used widely to transport genetic compositions owing to their excellent loading valence, and excellent transfection performance VLPs are non-infected virus-mimicking particles created via the self-assembly of protein monomers combined with viral capsid proteins [123]. Furthermore, the general impression of “nanoimmunity via design” can assist us to create substances for immune regulation, either inducing or inhibiting the immune reaction, which would find usages in the vaccine production for COVID-19 or in neutralizing the cytokine storm, in order [124]. Animal trials helped the technological comprehension of how these novel kinds of nanovaccines work. Furthermore, some of these nanovaccines candidates have entered clinical studies next to the initial assessment in animal models, of which NPs based vaccines are hopeful vaccine candidates. The Moderna and Pfizer SARS-CoV-2 infection vaccines, more than 90% efficient against COVID-19 in humans, apply the mRNA to generate the viral S protein found on the surface of SARS-CoV-2 [125, 126].

### Lipid-based nanoparticles vaccines

**Fig. 5:**
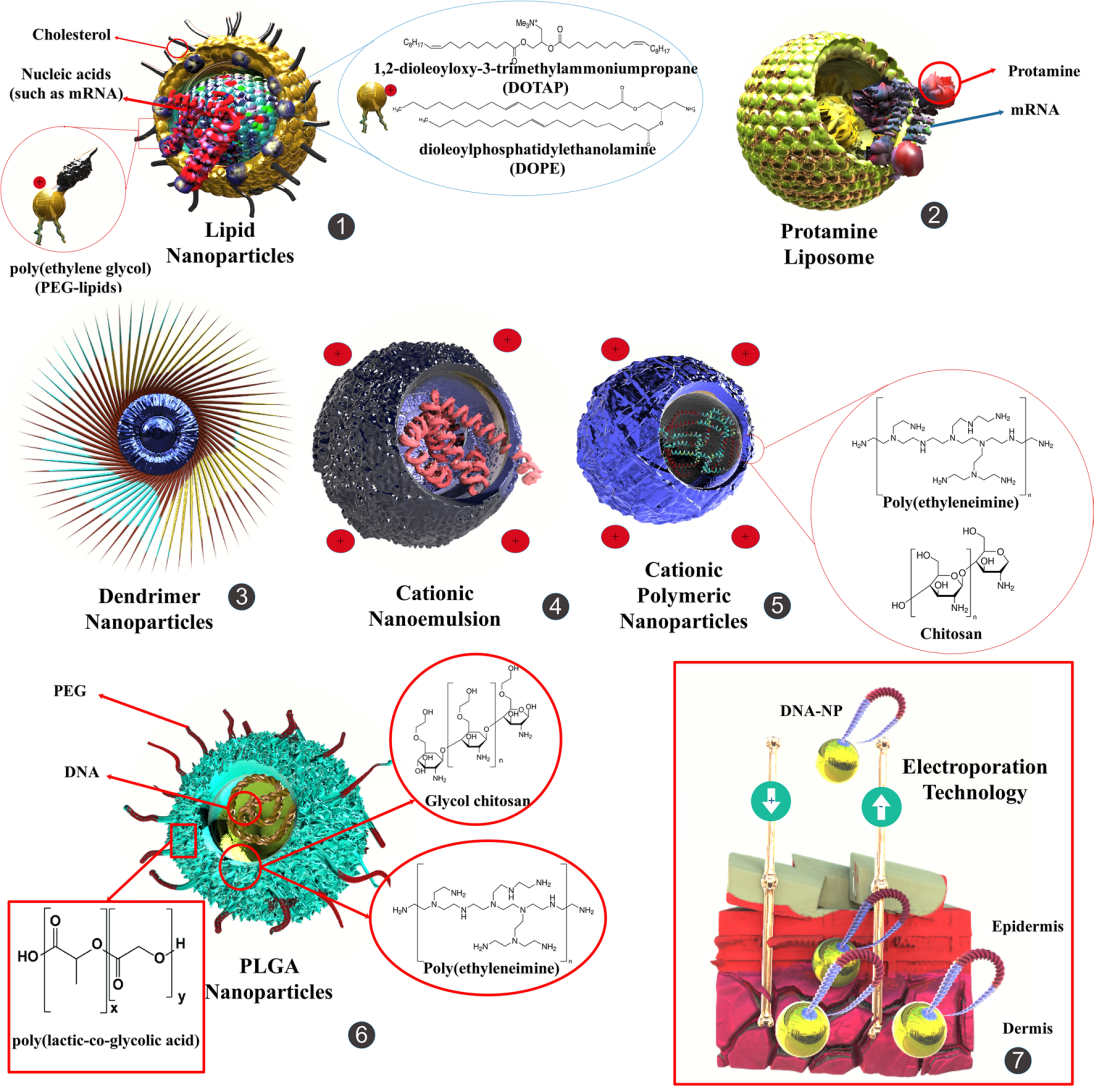
Several delivery methods for mRNA. LNPs are produced by the self-assembly of an ionizable cationic lipid. Various NPs of these cationic lipids (include 1,2-dioleoyloxy-3-trimethylammoniumpropane [DOTAP] or dioleoyl phosphatidylethanolamine [DOPE]) are prepared with subtle modifications (include cationic lipids + cholesterol NP, cationic lipids + cholesterol + PEG-LNP), where cholesterol and PEG-lipid are added to increase consistency. Other NP delivery systems include protamine (cationic peptide) nanoliposomes (sized approximately 100 nm), PEG-lipid functionalized dendrimer NPs (approximately 200 nm in size), positively charged oil-in-water (O/W) cationic nanoemulsion (approximately 120 nm in size), polyethyleneimine NP (approximately 100 − 300 nm in size), and cationic polymer (chitosan) NPs (approximately 300 − 600 nm in size)

Conventionally, mRNA has not been utilized as a remedial factor since it is greatly unconstant and triggers the innate immune responses when administered. In addition, to enter the host cells, mRNA needs a delivery system to pass the cell membrane [127]. Moreover, the average half-life of mRNA vaccines reduces with enhancing temperature, which is a problem for their long time storing. However, chemical alteration by using an external covering of nonionic or an ionic surfactant improves the thermal constancy of mRNA. These chemical modifications alter the sizes of a NP and help in the efficient carrying of mRNA with greater thermal constancy [128]. Consequently, researchers are developing NPs delivery system that contains LNPs that entrap the mRNAs coding for the monoclonal antibody. This RNA-treatment method steadies the mRNA and can be administered recurrently, causing sustained generation of antibodies evading the efficacy of the innate immunity versus exogenous RNA. Additionally, the LNPs increase their mucosal and cellular absorption. Moreover, the positively charged LNP results in electrostatic absorbency to the negative charge of the mucosal membranes, decreasing their release via the mucosal cilia [127]. For example, Moderna’s mRNA vaccine is based on an LNP method [129]. This method was newly proceeded via Moderna to generate vaccine methods versus COVID-19 via utilizing mRNA-1273, encoding viral S protein [130]. BNT162b2 is the first vaccine based on the LNP-mRNA method and mRNA encoding the S protein locked in its pre-fusion structure [131]. In other investigations, mRNA (RNActive®) based vaccines (CVnCoV), include sequence optimum mRNA coding to a fixed shape of S protein entrapped in LNP. No vaccine-associated acute side effects were detected. Immune reactions, when evaluated as IgG antibodies versus S protein or its RBD via ELISA, and COVID-19 neutralizing antibodies (NAbs) evaluated via micro-neutralization, showed dose-affiliate enhancement. Primary outcomes demonstrated that CVnCoV is as well as safe and excellent tolerated in tested individuals, and is capable of enhancing the pre-existing immune reaction even at fewer dosage amounts [132]. Other studies, developed self-amplifying RNA (saRNA) entrapped in LNP as a vaccine. These vaccine immunizations stimulate a Th1 biased reaction in the mouse model, and there is no antibody-related increase detected. In addition, they identified significant cellular responses, as determined via IFN-γ generation, upon re-stimulation by COVID-19 peptides [133]. Uri Elia et al. developed an mRNA vaccine, based on LNPs-entrapped COVID-19 virus human Fc-combined RBD (RBD-hFc). Intramuscular injection of this vaccine induced a strong humoral reaction, a significant amount of NAbs, and a Th1-biased cellular reaction in the BALB/c mouse model [134]. LNP-based vaccines (NVP) co-encapsulated by antigens and monophosphoryl lipid A (MPLA, a potent TLR-4 agonist) were easily absorbed via DCs and increased DC maturation and antigen offering. Multilamellar vesicles were formed via hydrating a lipid film combined with DOPC, n-(Succinimidyloxy-glutaryl)-L-α-phosphatidylethanolamine, dioleoyl (DOPE-NHS) and MPLA (50:50:0.5 molar proportion). Mice vaccinated through RBD-NVP triggered strong and persistent antibody reactions versus RBD from COVID-19 [135]. In another investigation, researchers developed quick transformation of recombinant RBD into particulate formation by incorporating with liposomes comprising cobalt-porphyrin-phospholipid (CoPoP) strongly improves the functional antibody reaction. Compared to other vaccine preparations, immunization utilizing CoPoP liposomes incorporated with recombinant RBD induces several orders of magnitude greater rates of antibody titers in mice that inhibit pseudovirus cell entrance, prevent RBD interaction with ACE2, and suppress SARS-CoV-2 replication [136] (Table 3, Fig. 5).

**Table 3:** Some significant examples of NP-based vaccine candidates versus SARS-CoV-2

NPs types	Developer	Study stage	Description	Refs.
LNP	BioNTech/Fosun Pharma/Pfizer	Approved in 141 countries and 70 clinical trials in 26 countries	The quick and greatly scalable mRNA are generating and LNP design procedures enable the fast generation of several vaccine dosages	[175]
LNP	Moderna/NIAID	Approved in 85 countries and 56 clinical trials in 22 countries	Encapsulate mRNA vaccines in LNPs, may allow cytoplasmic transfer via fusogenic. It is possible that they used formulations of ionizable lipid, DSPC, cholesterol, and polyethylene glycol-lipid	[178, 179]
LNP	Fudan University/Shanghai Jointing, University/RNAcare, Biopharma	Animal experiments	LNP‐entrapped mRNA cocktail encoding VLP	[146]
Lipid-based	Park, et al	Animal experiments	NVP co-encapsulated with antigens and monophosphoryl lipid A was readily taken up via DCs and increased DC maturation and antigen offer	[135]
Ferritin-based VLP	Ma et al	Animal experiments	RBD and RBD-HR NPs vaccines induce more powerful NAb reactions and T lymphocyte immune reactions than monomers	[139]
Mesoporous silica NPs	N4 Pharma Plc	Cellular studies	Nuvec® carrying nucleic acids have irregular surfaces functionalized with polyethyleneimine, and may be beneficial in the race to develop a COVID-19 vaccine	[140]
AuNPs	Sekimukai, et al	Animal experiments	AuNPs, which are expected to act as an antigen delivery systems and an adjuvant in immunization, and TLR agonists, which have formerly been shown to be an efficient adjuvant in an ultraviolet‐inactivated SARS‐CoV-2 vaccine	[180]
Recombinant NP	Novavax	Approved in 36 countries and 15 clinical trials in 12 countries	NVX-CoV2373 is a recombinant NP vaccine created from the full-length, wild-type COVID-19 virus S protein. It is joined with a saponin-based Matrix-M adjuvant to increasing the immune reaction and generating great amounts of NAbs, which are fundamental in inhibiting the disease	[142]
1c-SApNP	Ufovax	Phase I clinical trial	Automatic-mount protein NPs vaccine method (1c-SApNP) prototype offerings sections of COVID-19 virus proteins that protrude from a scaffold of protein NPs, targeting to incite the immune reaction and incite the creation of antibodies to inhibit COVID-19	[149]

### Virus-like particles vaccine

VLPs are multiprotein structures that mimic the organization and conformation of authentic native viruses but lack the viral genome, potentially yielding safer and cheaper vaccine candidates Ferritin as a naturally self-assembling protein nanoparticles is a favorite protein particle to develop VLP vaccines. The protein particles can be connected with a maximum of 24 viral antigens for concurrent delivery and offer to APCs. In recent studies, a developed subunit vaccine based on self-assembling ferritin NP showed one of two multimerized S proteins: full-length ectodomain (S-Fer) or a C-terminal 70 amino-acid deletion (SΔC-Fer). Mice vaccinated by one injection of S-Fer demonstrated remarkably more excellent NAbs titers than those immunized by RBD monomers or spike ectodomain trimers, suggesting the significance of multivalent exposure. Ferritin NPs conjugated with RBDs, instead of full-length S proteins, demonstrated a superior preservative immune reaction when contrasted with unconjugated RBDs [137]. In other investigations, researchers generated a VLP vaccine that exhibits 120 copies of SARS-CoV-2 RBD on its surface. This method mimics virus-based vaccines in immunogen present, which increases its effect while maintaining the lack of side effects of protein subunit vaccines. This vaccine elicited great-titer NAbs reactions in mice that continued for more than two months and powerfully suppressed COVID-19, SARS-CoV-1, and their variants [138]. Ma et al. created 2 Ferritin-based NP vaccines via conjugate RBD and heptad repeat (HR) antigens in S protein using the SpyTag/SpyCatcher method, which was obtained from *Streptococcus pyogenes*, to covalently bind the ferritin NPs instead of direct fusion expression, that the level of expression is significantly reduced. RBD and RBD-HR NPs vaccines induce more powerful NAb reactions and T lymphocyte immune reactions than monomers. HR-based NPs elicit cross-reactive immune reactions versus COVID-19 and other CoVs [139]. Medicago and iBio are utilizing Nicotiana benthamiana to generate VLPs using the S protein, and AdaptVac/ExpreS^2^ion is applying the insect cell expression method to create VLPs from the S2 protein. In addition, producing protein NPs from antigenic subunits, their expression and, or presentation on proteinaceous biomaterial scaffolds, including ferritin, encapsulin, and bacteriophage VLPs, has been used to attain multivalent antigen presentation for increased immunogenicity. The advantage of this method is that scalability and modularity; the peptides can be quickly modified as novel data concerning COVID-19, and its immunogenicity is made accessible; it is as well potential to adapt the method quickly should new or mutated kinds appear [129]. VLPs can be prepared via incubating AuNPs as a nucleus by CoV S proteins, which automatically functionalize the surface (S-AuNPs). S-AuNP-vaccines can improve lymphatic antigen transfer and enhance cellular and humoral reactions contrasted with free antigens. This nano vaccine was capable of eliciting a powerful IgG reaction; however, by a less desired to inhibit CoVs owing to alterations in the construction of S proteins upon connecting to AuNPs, leading to lung eosinophilic immunopathology [130].

### Other types of nanovaccines

To advance the creation of viral vaccines through mRNA and pDNA, a professional medicine company created a novel silica-based new technology called, Nuvec® designed for the transfer of vaccines and drugs. These new silica-NPs carrying nucleic acids have asymmetrical surfaces functionalized by polyethyleneimines (PEIs). This surface encapsulates nuclear acids (including mRNA/pDNA), as they cross into cells, and preserves them from nucleus enzymes. The critical benefit of Nuvec® is that it does not destroy the cell membrane when it attains the cells, contrasted to LNP; and not neither does it generate each inflammatory response at the administration location with no extreme systemic adverse events [140]. Peptide vaccines are known for their less immunogenicity, which could be dominated via combining immunostimulatory adjuvants and NPs, including PLGA or chitosan. The CD4 + and CD8 + T cell epitopes can as well be fused with adjuvant proteins such as TLR ligands which can be entrapped or presented on the surface of NPs such as PLGA to induce continued T-cell reactions and enhance long-range preservation. Smaller PLGA NPs (350 nm) allow improved internalization via DCs and cause a continued cellular immune reaction in mice. PLGA-NP encapsulated peptide antigens are being preserved against enzymatic destruction in vivo until they are absorbed via APCs and caused stimulation of strong B- and T-lymphocyte reactions [141]. In other investigations, SARS-CoV-2 vaccine developers, including GlaxoSmithKline, which owns the ASs and other adjuvant techniques, are employed in numerous companies to implant their adjuvant methods by COVID-19 virus-protein-based vaccines. The capability of approved adjuvants, including AS01 and AS03, to increase adaptive immune response has been related to their capacity to increase STAT1/IRF1 IFN signaling [124]. NVX-CoV2373 is a recombinant (rSARS-CoV-2) NP vaccine combined with trimeric full-length COVID-19 S proteins and Matrix-M1 adjuvant. Adjuvant led to increased immune reactions, was antigen dosage-sparing, and stimulated a Th1 response. The Matrix-M1 adjuvant elicited helper T lymphocytes reactions biased toward a Th1 phenotype. Matrix-M1, a saponin-based adjuvant, was created via Novavax. Both vaccine and adjuvant were maintained at 2–8 °C [142].

## Nanoparticles as a drug delivery system in SARS-CoV-2 infection

The nanocarrier of repurposed antiviral medicines can be improved by combining with cell-penetrating peptides (CPPs). CPPs transport the cargo within the cell either via macropinocytosis, caveolae-interceded endocytosis, or clathrin-free endocytosis manner. Therefore, the remedial use, of these CPPs should be fixed through the combination of multifunctional polymeric NPs or LNPs to increase selectivity, performance, and capacity of cargo delivery to prevent deactivation via proteases. So, nanoformulation-based CPP-encapsulated in NPs of repurposed antiviral medicines to treat SARS-CoV-2 infection. This method versus COVID-19 may be progressed and increased by conjugating it to the Tat-peptides via using nanoformulation-based NPs-delivery methods [143]. At present, siRNA treatment methods are inhibiting via the facility of siRNA enzymatic degradation, quick clearance, and lack of ability to pass into the cell membrane. However, these challenges can be notably addressed by utilizing NPs such as LNPs, polymeric NPs or their hybrid NPs, nanohydrogels, superparamagnetic iron-oxide NPs (SPIONs), and also functionalized AuNPs. PGA, poly(lactic acid) (PLA), polycaprolactone (PCL), and their copolymers PLGA have been accepted via FDA and more broadly utilized for in vivo siRNA transfer [144, 145]. As a result, the aerosol design for inhalation injection of designed siRNA NPs delivery system via measured-dosage inhaler is recommended as an effective way of an injection to the treatment of SARS-CoV-2 [146]. Inorganic polyphosphate (polyP), which was entrapped in silica/polyP NPs to inhibit polyP-destroying versus the alkaline phosphatase, was used to suppress connecting S protein to the ACE2 receptor in SARS-CoV-2. The result showed that completion of polyP might chip in improving the human innate immune responses in compromised, thrombocytopenic SARS-CoV-2 infected patients [147]. In other investigations, researchers developed recombinant DNase-1-covered PDA-poly(ethylene glycol) NPs (called long-acting DNase-1). The result showed that exogenously injected long-acting NPs DNase-1can efficiently decrease cell-free DNA (cfDNA) rates and neutrophil functions and may be utilized as powerful remedial intermediation for COVID-19. Researchers offered the remedial transport of a long-long-acting NP DNase-1 preparation for slowing the progress of sepsis in COVID-19 via inhibiting cfDNA [148].

Investigators have produced chitosan NPs for aerosol usage, which lets the adhesion and target delivery of medicines to the epithelial tissues of the lung and guarantees regulated discharge, thus decreasing the toxicity of the medications. Particular chitosan NP, named Novochizol, can entrapment of various medicines to deliver them to the lungs for the treatment of acute COVID-19 patients [149]. Other studies, used AgNPs, AuNPs, AgAu-NPs, and Pt NPs for delivery of the antiviral medicine, including hydroxychloroquine (HCQ), and chloroquine (CQ), to reduce the adverse events and increased the targeted therapy and efficacy of antiviral medications versus COVID-19 [150]. Moreover, dexamethasone encapsulated in NPs, and injecting it through intravenous or intranasal administration, can help to make better anti-SARS-CoV-2 therapy effect by targeting the strong corticosteroid medicine to hyper-triggered immune cells, with enhancing anti-edema acting and by eliciting its anti-fibrotic efficacy [151] (Table 4).

**Table 4:** Effects of different NPs as a delivery system and antiviral agent in SARS-COV-2 infection

NPs types	NPs performance generally	Description	Refs.
CQD	Antiviral attributes	Afterward, effective cell internalization and interaction with S protein, the NPs suppressed virus function against COVID-19	[155]
Zinc-based NPs	Antiviral attributes	These NPs suppress the mucosal binding of the virus and also prevent the virus reproduction and improve host immune reaction against viral infection	[155]
GO	Immune system inducer	These NPs stimulated IFN signaling triggering in T lymphocytes and monocytes. The generation of Th1-associated reaction has been displayed as critical for disease regulation in SARS-CoV-2	[155]
Nanodiamond	Antiviral responses	Octadecylamine-functionalized and dexamethasone-adsorbed nanodiamond decreased macrophage penetration and expression of proinflammatory mediator’s iNOS and TNF-α in the mouse	[124]
NO NPs	Antiviral attributes and therapeutic agent	These NPs can inhibit the beginning of inflammatory activities, decrease injuring vascular penetrance, and preserve sufficient blood circulation	[149]
IONPs	Antiviral attributes	These NPs interact with SARS-CoV-2 S1-RBD, which decreases infection	[159]
NC	Anti-inflammatory, antioxidant, and anti-fibrotic attributes	These NPs can per se reduce cytokine synthesis or inhibit function via inhibiting the receptor interaction of cytokines	[181]
Magnetic NPs	Antiviral attributes	Simultaneous, magnetic NPs interact with M-protein, leading to fragmentation of S protein	
Epithelial-NS, and MΦ-NS	Antiviral attributes	These nanosponges can inhibit the viral acting, and they will be capable of eliminating the severe inflammation dependent on SARS-CoV-2	[161]
Photothermal NPs	Virus binding inhibitor	These NPs showed effective capture of SARS-CoV-2, excellent photothermal efficacy, and complete suppression of viral entrance	[162]
TPNT1	Virus binding inhibitor	These NPs inhibit viral entrance via suppression of the binding of S proteins to ACE2 receptors	[163]
Silica/polyP NPs	Drug delivery system	The PolyP was entrapped in silica/polyP NPs to suppressing the connecting of S protein with ACE2	[147]
polydopamine-poly	Drug delivery system	Recombinant DNase-1-encapsulated polydopamine-poly	[148]
Novochizol™	Drug delivery system	These chitosan NPs can entrapment of various medicines to transport them to the lungs for the treatment of acute SARS-CoV-2	[149]
Ag, Au, AgAu, and Pt NPs	Drug delivery system and antiviral attributes	The noble metal NP as encouraging NPs with antiviral attributes, can deliver the HCQ and CQ to the target agent and reduce the adverse events	[150]

## Nanoparticles as antiviral agents against SARS-CoV-2 Infection

In the absence of carrying additional medicine payloads, NPs can use specific mechanisms for direct viral inhibiting. One of these mechanisms is the degradation of viral protein structure. Biocompatible NPs can demonstrate a wide range of antiviral actions. The action of specific NPs, such as AgNPs and AuNPs, can chip in the generally antiviral function [152].

Quantum dots (QD), a kind of crystalline NPs, have great nano-based detecting, and they, can be utilized as antiviral remedies. Various metallic combinations (Pb, Cu, Ga, Zn, Hg) based on QD displayed target particular activities versus viral infection [153]. Moreover, QDs can be employed as ideal options versus pathogenic human CoVs diseases; For example, the antiviral actions of 7 diverse carbon QDs (CQDs) for remedying HCoV-229E infections were assessed [154, 155]. Moreover, the available documentation shows that NPs, including graphene, nanodiamonds, carbon nanotubes, and polystyrene particles, possess an inherent capability to trigger the immune system, related to their functionalization. For example, graphene oxide functionalized with amino groups (GO-NH2) stimulates the triggering of STAT1/IRF1 IFN signaling in monocytes and T lymphocytes, leading to the generation of T cell chemoattractant, and macrophage 1/Th1 polarization of the immune reaction, with slight toxicity. Significantly, hypericin graphene is on the list of computationally recognized potential therapy versus SARS-CoV-2. Besides, graphene is a potent immunomodulator, and GO-AgNPs increase the generation of natural antiviral protections (IFN-α and ISGs) [156]. Octadecylamine-functionalized and dexamethasone-incorporated nanodiamonds increase anti-inflammatory and pro-regenerative performance in human macrophages in vitro. A less amount of this NP also decreased macrophage penetration and expression of proinflammatory intermediary’s inducible nitric oxide synthase (iNOS) and TNF-alpha in mice. Generally, outcomes showed that nanodiamond particles could be beneficial as an intrinsically immunomodulatory system [124]. The usage of nitric oxide NPs (NONPs) can also be an option in the therapy of SARS-CoV-2 disease. An investigation by SARS-CoV-1 detected that NO suppresses viral reproduction via the cytotoxic response from intermediate factors, including peroxynitrite. Since COVID-19 infects endothelial cells, which are an origin of NO, carrying NO from NPs may be an alternative for NO substitution, also a reaction to the viral targeting endothelial cells. Furthermore, suppressing viral prevalence, NO can inhibit the beginning of inflammatory activities based on hypoxia-reoxygenation /ischemia–reperfusion, regulator the cytokine cascade, let the elimination of cell fragments, restrict lipid peroxidation and cell injury, decrease damaging vascular penetrability, and preserve sufficient blood circulation [149]. Carbon nanotubes (CNT) belong to the fullerene family (sized 10–100 nm). Investigators developed a new method against COVID-19 by acidizing, and RALyase modified CNTs in combination with photodynamic thermal efficacy [157]. In another investigation, researchers hypothesized that carbon dots (CDs) derived from Allium sativum (AS-CDs) may have the capability to downregulate the expression of proinflammatory cytokines and return the immunological abnormalities to normal in SARS-CoV-2 infection. CDs have now been investigated in the nanobiomedicine field as a hopeful theranostic candidates for bioimaging and medication/gene transfer. The antifibrotic and antioxidant properties of AS are explained with accuracy, as confirmed in numerous investigations. It is found that the most active constituent of AS, allicin has an extremely strong antioxidant and reactive oxygen species (ROS) scavenging efficacy [158].

ZnNPs have been offered favorable versus the SARS-CoV-2 by preventing the mucosal connecting of the virus, inhibiting the virus reproduction, IFN-γ/α production, triggering the enzymes involved in several cellular actions, and reducing the inflammatory reaction, or increasing the immune system of the host [155]. Other investigations, showed that Iron oxide NPs (IONPs) (Fe2O3 and Fe3O4) interact with the S1-RBD COVID-19. Fe3O4 created a more constant composite with S1-RBD. These interplays of IONPs are anticipated to be related to viral protein structural alterations, and therefore viral inhibition. Consequently, recommend FDA-accepted-IONPs proceed with SARS-CoV-2 therapy clinical trials. Combinations that interact with the S1-RBD are hypothesized to inhibit virus binding to host receptors and prevent viral infection [159]. AuNPs can be excellent alternatives for antiviral factors versus SARS-CoV-2 infection. The capability of AuNPs functionalized via diverse groups, including 3-mercaptoe-ethyl sulfonate (Mes), undecanesulfonic acid (Mus), octanethiol (Ot), and a novel peptide, to suppress COVID-19 was studied. The results showed that functionalized AuNPs have significant influences on the RBD and powerfully interact with the virus protein. In addition, the AuNP functionalized via a novel peptide creates a more constant compound with RBD in contrast with ACE2. AuNP-EG2, AuNP-Ot, and AuNP-Pep coat the entire connection surface of RBD of the COVID-19 [160].

Zhang, et al. developed 2 kinds of cellular nanosponges, which are prepared of the plasma membranes derivative from human lung epithelial kind II cells (Epithelial-NS) or human macrophages (MΦ-NS) for the therapy of SARS-CoV-2. The membranes were then covered onto polymeric NPs cores developed from PLGA by a sonication technique to create Epithelial-NS and MΦ-NS, in order. Cell membrane covering lets nanosponges receive the viral receptors associated with CoVs entrance in the target cells; however, it will be capable of inhibiting the chronic inflammation related to SARS-CoV-2 infection [161]. Cai, et al. developed photothermal NP that includes a semiconducting polymer core, (PCPDTBT), which combines NAbs connected on the surface of a photothermal NP to catch and deactivate SARS-CoV-2 actively. An amphiphilic polymer cover is applied to entrap the PCPDTBT core. The NP, a biocompatible polyethylene glycol surface, is functionalized by a monoclonal NAb particular to the S protein, which allows targeted and effective catching of virus with great composite affinity (0.07 nM), thus inhibiting the entrance of virus into target cells. As soon as stimulation via a 650-nm light-emitting diode (LED), which has a more favorable harmlessness method than traditional laser stimulation, the photothermal NPs per se deactivate the caught COVID-19 via heat [162]. A metal NP compound TPNT1 (comprising AuNPs, AgNPs, ZnONPs, and Clo2) was able to suppress 6 main sections of SARS-CoV-2 by efficient concentration within the limited area as food additives. TPNT1 was shown to inhibit viral entrance via preventing the connection of S proteins to the ACE2 receptor and interacting with the syncytium form. As TPNT1 is most efficient via previous incubation with viruses, one potential mechanism for the antiviral action of TPNT1 may be ascribable to bindings of virus surface glycoproteins with the metal NPs, and so inhibiting the virions from binding to target cells [163] (Table 4).

## Conclusion

NPs can help to advance present treatments and as well be leveraged to generate new modalities which can destroy or prevent viruses via exclusive mechanisms of action. Based on their exclusive attributes, NPs have numerous specific benefits that can be leveraged to enhance the action of antiviral medicines. Payloads entrapped in NPs have less exposure to the exterior surroundings, which can help preserve them from systemic destruction while decreasing cytotoxicity. Moreover, NPs can increase the pharmacokinetic profiles of existing antiviral medicines via prolonging circulation time, binding particular tissue locations, and enhancing bioavailability. The efficiency of hydrophobic medications that are generally problematic to formulate and transport in vivo can be significantly improved by NPs. Owing to their perfect dimensions features and great surface area, NPs can associate with viruses in a multivalent method, allowing for extremely more powerful binding interactions. Generally, these developing therapeutics NPs are intrinsically resistant to viral mutations and can be used extensively versus a diversity of several viruses. NPs delivery methods contain antivirals, which can be produced from either synthetic or natural substances. Each one of these delivery methods has its collection of benefits and disadvantages for some uses. It is thus significant to choose the most suitable one and then improve its design. The SARS-CoV-2 pandemic continues to spread worldwide by an immediate requirement for a harmless and preservative vaccine to effectuate herd protection and regulate the spread of COVID-19. The rapid ratifications of mRNA1273 and BNT162b2 are significant achievements in the medication history. The NPs method can play a prime function in fighting SARS-CoV-2 using nanovaccines, which possess NPs and action as a delivery system of antigen that could trigger preservative immune responses. NPs have inherent immunomodulatory attributes that can affect the remedial act of vaccines. As the spread of viruses is faster than the development of effective vaccines, drug and vaccine studies should be complementary to what has already been achieved with previous CoV-related research.

## Data Availability

Not applicable.

## References

[1] Aghamirza H, Eivazzadeh-Keihan R, Beig A, Fattahi S, Maleki A, Fereshteh S, Bazaz M, Zolriasatein A, Bozorgnia B, Rahmati S (2022). COVID-19: A systematic review and update on prevention, diagnosis, and treatment. Med Comm.

[2] Yasamineh S, Kalajahi HG, Yasamineh P, Gholizadeh O, Youshanlouei HR, Matloub SK, Mozafari M, Jokar E, Yazdani Y, Dadashpour M (2022). Spotlight on therapeutic efficiency of mesenchymal stem cells in viral infections with a focus on COVID-19. Stem Cell Res Ther.

[3] Hashemi B, Akram F-A, Amirazad H, Dadashpour M, Sheervalilou M, Nasrabadi D, Ahmadi M, Sheervalilou R, Reza MAS, Ghazi F (2021). Emerging importance of nanotechnology-based approaches to control the COVID-19 pandemic; focus on nanomedicine iterance in diagnosis and treatment of COVID-19 patients. J Drug Deliv Sci Technol.

[4] Ahmadi K, Farasat A, Rostamian M, Johari B, Madanchi H (2022). Enfuvirtide, an HIV-1 fusion inhibitor peptide, can act as a potent SARS-CoV-2 fusion inhibitor: an in silico drug repurposing study. J Biomol Struct Dyn.

[5] Mousavi Maleki MS, Rostamian M, Madanchi H (2021). Antimicrobial peptides and other peptide-like therapeutics as promising candidates to combat SARS-CoV-2. Expert Rev Anti Infect Ther.

[6] Gallagher ME, Sieben AJ, Nelson KN, Kraay AN, Orenstein WA, Lopman B, Handel A, Koelle K (2021). Indirect benefits are a crucial consideration when evaluating SARS-CoV-2 vaccine candidates. Nat Med.

[7] Farshadi M, Johari B, Erfani Ezadyar E, Gholipourmalekabadi M, Azami M, Madanchi H, Haramshahi SMA, Yari A, Karimizade A, Nekouian R (2019). Nanocomposite scaffold seeded with mesenchymal stem cells for bone repair. Cell Biol Int.

[8] Nejati K, Rastegar M, Fathi F, Dadashpour M, Arabzadeh A (2022). Nanoparticle-based drug delivery systems to overcome gastric cancer drug resistance. J Drug Deliv Sci Technol.

[9] Yasamineh S, Yasamineh P, Kalajahi HG, Gholizadeh O, Yekanipour Z, Afkhami H, Eslami M, Kheirkhah AH, Taghizadeh M, Yazdani Y (2022). A state-of-the-art review on the recent advances of niosomes as a targeted drug delivery system. Int J Pharma.

[10] Kang J, Tahir A, Wang H, Chang J (2021). Applications of nanotechnology in virus detection, tracking, and infection mechanisms. Nanomed Nanobiotechnol.

[11] Nabizadeh Z, Nasrollahzadeh M, Daemi H, Eslaminejad MB, Shabani AA, Dadashpour M, Mirmohammadkhani M, Nasrabadi D (2022). Micro-and nanotechnology in biomedical engineering for cartilage tissue regeneration in osteoarthritis. Beilstein J Nanotechnol.

[12] Sahu AK, Sreepadmanabh M, Rai M, Chande A (2021). SARS-CoV-2: phylogenetic origins, pathogenesis, modes of transmission, and the potential role of nanotechnology. Virus Dis.

[13] Zamani R, Aval SF, Pilehvar-Soltanahmadi Y, Nejati-Koshki K, Zarghami N (2018). Recent advances in cell electrospining of natural and synthetic nanofibers for regenerative medicine. Drug Res.

[14] Chintagunta AD, Nalluru S (2021). Nanotechnology: an emerging approach to combat COVID-19. Emerg Mater.

[15] Yousefi B, Valizadeh S, Ghaffari H, Vahedi A, Karbalaei M, Eslami M. A global treatments for coronaviruses including COVID‐19. J Cell Physiol. 2020;235(12):9133–42. 10.1002/jcp.29785 PMC7273044 32394467

[16] Yousefi B, Banihashemian SZ, Feyzabadi ZK, Hasanpour S, Kokhaei P, Abdolshahi A, Emadi A, Eslami M. Potential therapeutic effect of oxygen-ozone in controlling of COVID-19 disease. Med Gas Res. 2022;12(2):33. 10.4103/2045-9912.325989 PMC8562402 34677149

[17] Chan JF, Lau SK, To KK, Cheng VC, Woo PC, Yuen K-Y (2015). Middle East respiratory syndrome coronavirus: another zoonotic betacoronavirus causing SARS-like disease. Clin Microbiol Rev.

[18] Wang Q, Zhang Y, Wu L, Niu S, Song C, Zhang Z, Lu G, Qiao C, Hu Y, Yuen K-Y (2020). Structural and functional basis of SARS-CoV-2 entry by using human ACE2. Cell.

[19] Hu B, Guo H, Zhou P, Shi Z-L (2020). Characteristics of SARS-CoV-2 and COVID-19. Nat Rev Microbiol.

[20] Letko M, Marzi A, Munster V (2020). Functional assessment of cell entry and receptor usage for SARS-CoV-2 and other lineage B betacoronaviruses. Nat Microbiol.

[21] Saberiyan M, Safi A, Kamel A, Movahhed-Abbasabad P, Miralimalek M, Afkhami H, Khaledi M, Teimori H (2020). An Overview on the Common Laboratory Parameter Alterations and their Related Molecular Pathways in Screening for COVID-19 Patients. Clin Lab.

[22] Singh V, Allawadhi P, Khurana A, Banothu AK, Bharani KK (2021). Critical neurological features of COVID-19: Role of imaging methods and biosensors for effective diagnosis. Sens Int.

[23] Yousefi B, Eslami M. Genetic and structure of novel coronavirus COVID-19 and molecular mechanisms in the pathogenicity of coronaviruses. Rev Med Microbiol. 2022;33(1):e180-8.

[24] Islam MR, Hoque MN, Rahman MS, Alam ARU, Akther M, Puspo JA, Akter S, Sultana M, Crandall KA, Hossain MA (2020). Genome-wide analysis of SARS-CoV-2 virus strains circulating worldwide implicates heterogeneity. Sci Rep.

[25] Khaledi M, Yousefi Nojookambari N, Afkhami H, Sameni F, Yazdansetad S (2021). A review on phylogenetic assessment and cytopathogenesis of filoviruses, retroviruses, and coronaviruses transmitted from bat to human. Cell Mol Res.

[26] Kim D, Lee J-Y, Yang J-S, Kim JW, Kim VN, Chang H (2020). The architecture of SARS-CoV-2 transcriptome. Cell.

[27] Khailany RA, Safdar M, Ozaslan M (2020). Genomic characterization of a novel SARS-CoV-2. Gene reports.

[28] Zhang L, Lin D, Sun X, Curth U, Drosten C, Sauerhering L, Becker S, Rox K, Hilgenfeld R (2020). Crystal structure of SARS-CoV-2 main protease provides a basis for design of improved α-ketoamide inhibitors. Science.

[29] Yao H, Song Y, Chen Y, Wu N, Xu J, Sun C, Zhang J, Weng T, Zhang Z, Wu Z (2020). Molecular architecture of the SARS-CoV-2 virus. Cell.

[30] Bestle D, Heindl MR, Limburg H, Pilgram O, Moulton H, Stein DA, Hardes K, Eickmann M, Dolnik O, Rohde C (2020). TMPRSS2 and furin are both essential for proteolytic activation of SARS-CoV-2 in human airway cells. Life Science Alliance.

[31] Hosseini P, Rahimi H, Najafabadi MM, Ghorbani A, Najafabadi SK, Faridzadeh A, Arabpour J, Khormali E, Deravi N (2021). Convalescent plasma therapy for COVID-19: lessons from SARS-CoV, MERS-CoV, and H1N1 infection. Infection.

[32] Branson B, Tavakoli R, Khaledi M, Shafiee SM, Afkham H, Rastegar S (2021). The Correlations Between Epidemiological and Clinical Characteristics, laboratory tests and CT Scan reports in the diagnosis of cases 2019 novel coronavirus pneumonia. A Diagnostic Accuracy Study. Authorea Preprints.

[33] Ke Z, Oton J, Qu K, Cortese M, Zila V, McKeane L, Nakane T, Zivanov J, Neufeldt CJ, Cerikan B (2020). Structures and distributions of SARS-CoV-2 spike proteins on intact virions. Nature.

[34] Charelli LE, de Mattos GC, de Jesus S-B, Pinto JC, Balbino TA (2022). Polymeric nanoparticles as therapeutic agents against coronavirus disease. J Nanopart Res.

[35] Amirsaadat S, Jafari-Gharabaghlou D, Alijani S, Mousazadeh H, Dadashpour M, Zarghami N (2021). Metformin and Silibinin co-loaded PLGA-PEG nanoparticles for effective combination therapy against human breast cancer cells. J Drug Deliv Sci Technol.

[36] Samadzadeh S, Mousazadeh H, Ghareghomi S, Dadashpour M, Babazadeh M, Zarghami N (2021). In vitro anticancer efficacy of Metformin-loaded PLGA nanofibers towards the post-surgical therapy of lung cancer. J Drug Deliv Sci Technol.

[37] Sahdev P, Ochyl LJ, Moon JJ (2014). Biomaterials for nanoparticle vaccine delivery systems. Pharm Res.

[38] de Souza GAP, Rocha RP, Gonçalves RL, Ferreira CS, de Mello SB, de Castro RFG, Rodrigues JFV, Júnior JCVV, Malaquias LCC, Abrahão JS (2021). Nanoparticles as vaccines to prevent arbovirus infection: a long road ahead. Pathogens.

[39] Abo-zeid Y, Garnett MC (2020). Polymer nanoparticle as a delivery system for ribavirin: do nanoparticle avoid uptake by Red Blood Cells?. J Drug Deliv Sci Technol.

[40] Chen L, Liang J (2020). An overview of functional nanoparticles as novel emerging antiviral therapeutic agents. Mater Sci Eng, C.

[41] Chen N, Zheng Y, Yin J, Li X, Zheng C (2013). Inhibitory effects of silver nanoparticles against adenovirus type 3 in vitro. J Virol Methods.

[42] Jafari-Gharabaghlou D, Pilehvar-Soltanahmadi Y, Dadashpour M, Mota A, Vafajouy-Jamshidi S, Faramarzi L, Rasouli S, Zarghami N (2018). Combination of metformin and phenformin synergistically inhibits proliferation and hTERT expression in human breast cancer cells. Iran J Basic Med Sci.

[43] Javan N, Khadem Ansari MH, Dadashpour M, Khojastehfard M, Bastami M, Rahmati-Yamchi M, Zarghami N (2019). Synergistic antiproliferative effects of co-nanoencapsulated curcumin and chrysin on mda-mb-231 breast cancer cells through upregulating mir-132 and mir-502c. Nutr Cancer.

[44] Delshadi R, Bahrami A, McClements DJ, Moore MD, Williams L (2021). Development of nanoparticle-delivery systems for antiviral agents: A review. J Control Release.

[45] Gurunathan S, Qasim M, Choi Y, Do JT, Park C, Hong K, Kim J-H, Song H (2020). Antiviral potential of nanoparticles—Can nanoparticles fight against coronaviruses?. Nanomaterials.

[46] Pourgholi A, Dadashpour M, Mousapour A, Amandi AF, Zarghami N (2021). Anticancer potential of silibinin loaded polymeric nanoparticles against breast cancer cells: insight into the apoptotic genes targets. Asian Pac J Cancer Prevent.

[47] Dadashpour M, Ganjibakhsh M, Mousazadeh H, Nejati K (2022). Increased pro-apoptotic and anti-proliferative activities of simvastatin encapsulated PCL-PEG Nanoparticles on human breast cancer adenocarcinoma cells. J Cluster Sci.

[48] Surnar B, Kamran MZ, Shah AS, Basu U, Kolishetti N, Deo S, Jayaweera DT, Daunert S, Dhar S (2019). Orally administrable therapeutic synthetic nanoparticle for Zika virus. ACS Nano.

[49] Zhang G, Campbell GR, Zhang Q, Maule E, Hanna J, Gao W, Zhang L, Spector SA (2020). CD4+ t cell-mimicking nanoparticles broadly neutralize hiv-1 and suppress viral replication through autophagy. MBio.

[50] Wei X, Zhang G, Ran D, Krishnan N, Fang RH, Gao W, Spector SA, Zhang L (2018). T-Cell-mimicking nanoparticles can neutralize HIV Infectivity. Adv Mater.

[51] Sanna V, Satta S, Hsiai T, Sechi M (2022). Development of targeted nanoparticles loaded with antiviral drugs for SARS-CoV-2 inhibition. Eur J Med Chem.

[52] Khater SE, El-Khouly A, Abdel-Bar HM, Al-Mahallawi AM, Ghorab DM (2021). Fluoxetine hydrochloride loaded lipid polymer hybrid nanoparticles showed possible efficiency against SARS-CoV-2 infection. Int J Pharm.

[53] Alavi M, Karimi N, Safaei M (2017). Application of various types of liposomes in drug delivery systems. Adv Pharm Bull.

[54] Aguilera-Correa JJ, Esteban J, Vallet-Regí M (2021). Inorganic and polymeric nanoparticles for human viral and bacterial infections prevention and treatment. Nanomaterials.

[55] Thi EP, Mire CE, Lee AC, Geisbert JB, Zhou JZ, Agans KN, Snead NM, Deer DJ, Barnard TR, Fenton KA (2015). Lipid nanoparticle siRNA treatment of Ebola-virus-Makona-infected nonhuman primates. Nature.

[56] Wang J, Li P, Yu Y, Fu Y, Jiang H, Lu M, Sun Z, Jiang S, Lu L, Wu MX (2020). Pulmonary surfactant–biomimetic nanoparticles potentiate heterosubtypic influenza immunity. Science.

[57] Huang H, Zhang C, Yang S, Xiao W, Zheng Q, Song X (2021). The investigation of mRNA vaccines formulated in liposomes administrated in multiple routes against SARS-CoV-2. J Control Release.

[58] Wang J, Yin X-G, Wen Y, Lu J, Zhang R-Y, Zhou S-H, Liao C-M, Wei H-W, Guo J (2022). MPLA-Adjuvanted Liposomes Encapsulating S-Trimer or RBD or S1, but Not S-ECD, Elicit Robust Neutralization Against SARS-CoV-2 and Variants of Concern. J Med Chem.

[59] Thipparaboina R, Chavan RB, Kumar D, Modugula S, Shastri NR (2015). Micellar carriers for the delivery of multiple therapeutic agents. Colloids Surf, B.

[60] Varela-Garcia A, Concheiro A, Alvarez-Lorenzo C (2018). Soluplus micelles for acyclovir ocular delivery: Formulation and cornea and sclera permeability. Int J Pharm.

[61] Koppisetti RK, Fulcher YG, Van Doren SR (2021). Fusion peptide of SARS-CoV-2 spike rearranges into a wedge inserted in bilayered micelles. J Am Chem Soc.

[62] Li Q, Huang Q, Kang C (2022). Secondary Structures of the Transmembrane Domain of SARS-CoV-2 Spike Protein in Detergent Micelles. Int J Mol Sci.

[63] Dias AP, da Silva SS, da Silva JV, Parise-Filho R, Ferreira EI, El Seoud O, Giarolla J (2020). Dendrimers in the context of nanomedicine. Int J Pharm.

[64] Kandeel M, Al-Taher A, Park BK, Kwon HJ, Al-Nazawi M (2020). A pilot study of the antiviral activity of anionic and cationic polyamidoamine dendrimers against the Middle East respiratory syndrome coronavirus. J Med Virol.

[65] Sepúlveda-Crespo D, Jiménez JL, Gómez R, De La Mata FJ, Majano PL, Muñoz-Fernández MÁ, Gastaminza P (2017). Polyanionic carbosilane dendrimers prevent hepatitis C virus infection in cell culture. Nanomedicine.

[66] Khaitov M, Nikonova A, Shilovskiy I, Kozhikhova K, Kofiadi I, Vishnyakova L, Nikolskii A, Gattinger P, Kovchina V, Barvinskaia E (2021). Silencing of SARS-CoV-2 with modified siRNA-peptide dendrimer formulation. Allergy.

[67] Mignani S, Shi X, Karpus A, Lentini G, Majoral J-P (2021). Functionalized dendrimer platforms as a new forefront arsenal targeting SARS-CoV-2: An Opportunity. Pharmaceutics.

[68] Paliwal R, Paliwal SR, Kenwat R, Kurmi BD, Sahu MK (2020). Solid lipid nanoparticles: a review on recent perspectives and patents. Expert Opin Ther Pat.

[69] Kondel R, Shafiq N, Kaur IP, Singh MP, Pandey AK, Ratho RK, Malhotra S (2019). Effect of Acyclovir Solid Lipid Nanoparticles for the Treatment of Herpes Simplex Virus (HSV) Infection in an Animal Model of HSV-1 Infection. Pharma Nanotechnol.

[70] Tulbah AS, Lee W-H (2021). Physicochemical Characteristics and In Vitro Toxicity/Anti-SARS-CoV-2 Activity of Favipiravir Solid Lipid Nanoparticles (SLNs). Pharmaceuticals.

[71] Fulcher JA, Tamshen K, Wollenberg AL, Kickhoefer VA, Mrazek J, Elliott J, Ibarrondo FJ, Anton PA, Rome LH, Maynard HD (2019). Human vault nanoparticle targeted delivery of antiretroviral drugs to inhibit human immunodeficiency virus type 1 infection. Bioconjug Chem.

[72] Rungrojcharoenkit K, Sunintaboon P, Ellison D, Macareo L, Midoeng P, Chaisuwirat P, Fernandez S, Ubol S (2020). Development of an adjuvanted nanoparticle vaccine against influenza virus, an in vitro study. PLoS ONE.

[73] Lauster D, Klenk S, Ludwig K, Nojoumi S, Behren S, Adam L, Stadtmüller M, Saenger S, Zimmler S, Hönzke K (2020). Phage capsid nanoparticles with defined ligand arrangement block influenza virus entry. Nat Nanotechnol.

[74] Gunathilake TMSU, Ching YC, Uyama H, Hai ND, Chuah CH (2022). Enhanced curcumin loaded nanocellulose: a possible inhalable nanotherapeutic to treat COVID-19. Cellulose.

[75] Hanafy NA, El-Kemary MA (2022). Silymarin/curcumin loaded albumin nanoparticles coated by chitosan as muco-inhalable delivery system observing anti-inflammatory and anti COVID-19 characterizations in oleic acid triggered lung injury and in vitro COVID-19 experiment. Int J Biol Macromol.

[76] Poon C, Patel AA (2020). Organic and inorganic nanoparticle vaccines for prevention of infectious diseases. Nano Express.

[77] Maleki MJ, Ghasemi Y, Pourhassan-Moghaddam M, Asadi N, Dadashpour M, Mohammadi SA, Akbarzadeh A, Zarghami N (2019). Effect of green GO/Au nanocomposite on in-vitro amplification of human DNA. IET Nanobiotechnol.

[78] Nejati K, Dadashpour M, Gharibi T, Mellatyar H, Akbarzadeh A (2021). Biomedical applications of functionalized gold nanoparticles: a review. J Cluster Sci.

[79] Ferrando RM, Lay L, Polito L (2021). Gold nanoparticle-based platforms for vaccine development. Drug Discovery Today.

[80] Lee M-Y, Yang J-A, Jung HS, Beack S, Choi JE, Hur W, Koo H, Kim K, Yoon SK, Hahn SK (2012). Hyaluronic acid–gold nanoparticle/interferon α complex for targeted treatment of hepatitis C virus infection. ACS Nano.

[81] Halder A, Das S, Ojha D, Chattopadhyay D, Mukherjee A (2018). Highly monodispersed gold nanoparticles synthesis and inhibition of herpes simplex virus infections. Mater Sci Eng, C.

[82] Farfán-Castro S, García-Soto MJ, Comas-García M, Arévalo-Villalobos JI, Palestino G, González-Ortega O, Rosales-Mendoza S (2021). Synthesis and immunogenicity assessment of a gold nanoparticle conjugate for the delivery of a peptide from SARS-CoV-2. Nanomedicine.

[83] Chen X, Han W, Wang G, Zhao X (2020). Application prospect of polysaccharides in the development of anti-novel coronavirus drugs and vaccines. Int J Biol Macromol.

[84] Dadashpour M, Firouzi-Amandi A, Pourhassan-Moghaddam M, Maleki MJ, Soozangar N, Jeddi F, Nouri M, Zarghami N, Pilehvar-Soltanahmadi Y (2018). Biomimetic synthesis of silver nanoparticles using Matricaria chamomilla extract and their potential anticancer activity against human lung cancer cells. Mater Sci Eng C.

[85] Pilaquinga F, Morey J, Torres M, Seqqat R (2021). Piña MdLN: Silver nanoparticles as a potential treatment against SARS-CoV-2: A review. Nanomed Nanobiotechnol.

[86] Allawadhi P, Singh V, Khurana A, Khurana I, Allwadhi S, Kumar P, Banothu AK, Thalugula S, Barani PJ, Naik RR (2021). Silver nanoparticle based multifunctional approach for combating COVID-19. Sens Int.

[87] Xiang D, Zheng Y, Duan W, Li X, Yin J, Shigdar S, O’Connor ML, Marappan M, Zhao X, Miao Y (2013). Inhibition of A/Human/Hubei/3/2005 (H3N2) influenza virus infection by silver nanoparticles in vitro and in vivo. Int J Nanomed.

[88] Wan C, Tai J, Zhang J, Guo Y, Zhu Q, Ling D, Gu F, Gan J, Zhu C, Wang Y (2019). Silver nanoparticles selectively induce human oncogenic γ-herpesvirus-related cancer cell death through reactivating viral lytic replication. Cell Death Dis.

[89] Almanza-Reyes H, Moreno S, Plascencia-López I, Alvarado-Vera M, Patrón-Romero L, Borrego B, Reyes-Escamilla A, Valencia-Manzo D, Brun A, Pestryakov A (2021). Evaluation of silver nanoparticles for the prevention of SARS-CoV-2 infection in health workers: In vitro and in vivo. PLoS ONE.

[90] Al-Sanea MM, Abelyan N, Abdelgawad MA, Musa A, Ghoneim MM, Al-Warhi T, Aljaeed N, Alotaibi OJ, Alnusaire TS, Abdelwahab SF (2021). Strawberry and ginger silver nanoparticles as potential inhibitors for SARS-CoV-2 assisted by in silico modeling and metabolic profiling. Antibiotics.

[91] AbouAitah K, Swiderska-Sroda A, Kandeil A, Salman AM, Wojnarowicz J, Ali MA, Opalinska A, Gierlotka S, Ciach T, Lojkowski W (2020). Virucidal Action Against Avian Influenza H5N1 virus and immunomodulatory effects of nanoformulations consisting of mesoporous silica nanoparticles loaded with natural prodrugs. Int J Nanomed.

[92] Bimbo LM, Denisova OV, Mäkilä E, Kaasalainen M, De Brabander JK, Hirvonen J, Salonen J, Kakkola L, Kainov D, Santos HA (2013). Inhibition of influenza A virus infection in vitro by saliphenylhalamide-loaded porous silicon nanoparticles. ACS Nano.

[93] Agelidis A, Koujah L, Suryawanshi R, Yadavalli T, Mishra YK, Adelung R, Shukla D (2019). An intra-vaginal zinc oxide tetrapod nanoparticles (zoten) and genital herpesvirus cocktail can provide a novel platform for live virus vaccine. Front Immunol.

[94] Norouzi M, Yasamineh S, Montazeri M, Dadashpour M, Sheervalilou R, Abasi M, Pilehvar-Soltanahmadi Y (2019). Recent advances on nanomaterials-based fluorimetric approaches for microRNAs detection. Mater Sci Eng C.

[95] Kumar R, Nayak M, Sahoo GC, Pandey K, Sarkar MC, Ansari Y, Das V, Topno R, Madhukar M, Das P (2019). Iron oxide nanoparticles based antiviral activity of H1N1 influenza A virus. J Infect Chemother.

[96] Sarkar PK, Das Mukhopadhyay C (2021). Ayurvedic metal nanoparticles could be novel antiviral agents against SARS-CoV-2. International Nano Letters.

[97] Wang J, Yu Y, Leng T, Li Y, Lee S-T (2021). The Inhibition of SARS-CoV-2 3CL Mpro by graphene and its derivatives from molecular dynamics simulations. ACS Appl Mater Interfaces.

[98] Li Y, Shi X (2013). MicroRNAs in the regulation of TLR and RIG-I pathways. Cell Mol Immunol.

[99] Hu J, Stojanović J, Yasamineh S, Yasamineh P, Karuppannan SK, Dowlath MJH, Serati-Nouri H (2021). The potential use of microRNAs as a therapeutic strategy for SARS-CoV-2 infection. Arch Virol.

[100] Lei X, Dong X, Ma R, Wang W, Xiao X, Tian Z, Wang C, Wang Y, Li L, Ren L (2020). Activation and evasion of type I interferon responses by SARS-CoV-2. Nat Commun.

[101] Portela C, Brites C (2020). Immune response in SARS-CoV-2 infection: the role of interferons type I and type III. Brazil J Infect Dis.

[102] Tufan A (2020). COVID-19, immune system response, hyperinflammation and repurposing antirheumatic drugs. Turkish J Med Sci.

[103] Izadi M, Tahmasebi S, Pustokhina I, Yumashev AV, Lakzaei T, Alvanegh AG, Roshangar L, Dadashpour M, Yousefi M, Ahmadi M (2020). Changes in Th17 cells frequency and function after ozone therapy used to treat multiple sclerosis patients. Multiple Sclerosis Related Disorders.

[104] Ahmadpoor P, Rostaing L (2020). Why the immune system fails to mount an adaptive immune response to a Covid-19 infection. Transplant Int.

[105] Tu Y-F, Chien C-S, Yarmishyn AA, Lin Y-Y, Luo Y-H, Lin Y-T, Lai W-Y, Yang D-M, Chou S-J, Yang Y-P (2020). A review of SARS-CoV-2 and the ongoing clinical trials. Int J Mol Sci.

[106] Hoffmann M, Kleine-Weber H, Schroeder S, Krüger N, Herrler T, Erichsen S, Schiergens TS, Herrler G, Wu N-H, Nitsche A (2020). SARS-CoV-2 cell entry depends on ACE2 and TMPRSS2 and is blocked by a clinically proven protease inhibitor. Cell.

[107] Chen C, Huang J, Cheng Z, Wu J, Chen S, Zhang Y, Chen B, Lu M, Luo Y, Zhang J (2020). Favipiravir versus Arbidol for COVID-19: A Randomized Clinical Trial. MedRxiv.

[108] Ko W-C, Rolain J-M, Lee N-Y, Chen P-L, Huang C-T, Lee P-I, Hsueh P-R (2020). Arguments in favour of remdesivir for treating SARS-CoV-2 infections. Int J Antimicrob Agents.

[109] Wang T, Du Z, Zhu F, Cao Z, An Y, Gao Y, Jiang B (2020). Comorbidities and multi-organ injuries in the treatment of COVID-19. Lancet.

[110] Zhang H, Penninger JM, Li Y, Zhong N, Slutsky AS (2020). Angiotensin-converting enzyme 2 (ACE2) as a SARS-CoV-2 receptor: molecular mechanisms and potential therapeutic target. Intensive Care Med.

[111] Zhang B, Liu S, Tan T, Huang W, Dong Y, Chen L, Chen Q, Zhang L, Zhong Q, Zhang X (2020). Treatment with convalescent plasma for critically ill patients with SARS-CoV-2 infection. Chest.

[112] Khurana I, Allawadhi P, Khurana A, Srivastava AK, Navik U, Banothu AK, Bharani KK (2021). Can bilirubin nanomedicine become a hope for the management of COVID-19?. Med Hypotheses.

[113] Krammer F (2020). SARS-CoV-2 vaccines in development. Nature.

[114] Wang H, Zhang Y, Huang B, Deng W, Quan Y, Wang W, Xu W, Zhao Y, Li N, Zhang J (2020). Development of an inactivated vaccine candidate, BBIBP-CorV, with potent protection against SARS-CoV-2. Cell.

[115] Gao Q, Bao L, Mao H, Wang L, Xu K, Yang M, Li Y, Zhu L, Wang N, Lv Z (2020). Development of an inactivated vaccine candidate for SARS-CoV-2. Science.

[116] Creech CB, Walker SC, Samuels RJ (2021). SARS-CoV-2 vaccines. JAMA.

[117] Chavda VP, Hossain MK, Beladiya J, Apostolopoulos V (2021). Nucleic acid vaccines for COVID-19: a paradigm shift in the vaccine development arena. Biologics.

[118] Dzinamarira T, Tungwarara N, Chitungo I, Chimene M, Iradukunda PG, Mashora M, Murewanhema G, Rwibasira GN, Musuka G (2022). Unpacking the Implications of SARS-CoV-2 Breakthrough Infections on COVID-19 Vaccination Programs. Vaccines.

[119] Krammer F (2020). SARS-CoV-2 vaccines in development. Nature.

[120] Li Y, Tenchov R, Smoot J, Liu C, Watkins S, Zhou Q (2021). A comprehensive review of the global efforts on COVID-19 vaccine development. ACS Cent Sci.

[121] Chen W-H, Strych U, Hotez PJ, Bottazzi ME (2020). The SARS-CoV-2 vaccine pipeline: an overview. Curr Trop Med Rep.

[122] Singh L, Kruger HG, Maguire GE, Govender T, Parboosing R (2017). The role of nanotechnology in the treatment of viral infections. Ther Advan Infect Dis.

[123] Duan Y, Wang S, Zhang Q, Gao W, Zhang L (2021). Nanoparticle approaches against SARS-CoV-2 infection. Curr Opin Solid State Mater Sci.

[124] Weiss C, Carriere M, Fusco L, Capua I, Regla-Nava JA, Pasquali M, Scott JA, Vitale F, Unal MA, Mattevi C (2020). Toward Nanotechnology-Enabled Approaches against the COVID-19 Pandemic. ACS Nano.

[125] Zhang N-N, Zhang R-R, Zhang Y-F, Ji K, Xiong X-C, Qin Q-S, Gao P, Lu X-S, Zhou H-Y, Song H-F (2022). Rapid development of an updated mRNA vaccine against the SARS-CoV-2 Omicron variant. Cell Res.

[126] Sun J, Zheng Q, Madhira V, Olex AL, Anzalone AJ, Vinson A, Singh JA, French E, Abraham AG, Mathew J (2022). Association between immune dysfunction and COVID-19 breakthrough infection after SARS-CoV-2 vaccination in the US. JAMA Intern Med.

[127] Ruiz-Hitzky E, Darder M, Wicklein B, Ruiz-Garcia C, Martín-Sampedro R, Del Real G, Aranda P (2020). Nanotechnology Responses to COVID-19. Adv Healthcare Mater.

[128] Khurana A, Allawadhi P, Khurana I, Allwadhi S, Weiskirchen R, Banothu AK, Chhabra D, Joshi K, Bharani KK (2021). Role of nanotechnology behind the success of mRNA vaccines for COVID-19. Nano Today.

[129] Shin MD, Shukla S, Chung YH, Beiss V, Chan SK, Ortega-Rivera OA, Wirth DM, Chen A, Sack M, Pokorski JK (2020). COVID-19 vaccine development and a potential nanomaterial path forward. Nat Nanotechnol.

[130] Abd Ellah NH, Gad SF, Muhammad K (2020). Nanomedicine as a promising approach for diagnosis, treatment and prophylaxis against COVID-19. Nanomedicine.

[131] Bowman CJ, Bouressam M, Campion SN, Cappon GD, Catlin NR, Cutler MW, Diekmann J, Rohde CM, Sellers RS, Lindemann C (2021). Lack of effects on female fertility and prenatal and postnatal offspring development in rats with BNT162b2, a mRNA-based COVID-19 vaccine. Reprod Toxicol.

[132] Kremsner P, Mann P, Bosch J, Fendel R, Gabor JJ, Kreidenweiss A, Kroidl A, Leroux-Roels I, Leroux-Roels G, Schindler C: Phase 1 Assessment of the Safety and Immunogenicity of an mRNA-Lipid Nanoparticle Vaccine Candidate Against SARS-CoV-2 in Human Volunteers. medRxiv 2020. 10.1007/s00508-021-01922-y PMC8354521 34378087

[133] McKay PF, Hu K, Blakney AK, Samnuan K, Brown JC, Penn R, Zhou J, Bouton CR, Rogers P, Polra K (2020). Self-amplifying RNA SARS-CoV-2 lipid nanoparticle vaccine candidate induces high neutralizing antibody titers in mice. Nat Commun.

[134] Elia U, Ramishetti S, Rosenfeld R, Dammes N, Bar-Haim E, Naidu GS, Makdasi E, Yahalom-Ronen Y, Tamir H, Paran N (2021). Design of SARS-CoV-2 hFc-Conjugated Receptor-Binding Domain mRNA Vaccine Delivered via Lipid Nanoparticles. ACS Nano.

[135] Park KS, Bazzill JD, Son S, Nam J, Shin SW, Ochyl LJ, Stuckey JA, Meagher JL, Chang L, Song J (2020). Lipid-based vaccine nanoparticles for induction of humoral immune responses against HIV-1 and SARS-CoV-2. J Controlled Release.

[136] Huang WC, Zhou S, He X, Chiem K, Mabrouk MT, Nissly RH, Bird IM, Strauss M, Sambhara S, Ortega J (2020). SARS-CoV-2 RBD neutralizing antibody induction is enhanced by particulate vaccination. Adv Mater.

[137] Powell AE, Zhang K, Sanyal M, Tang S, Weidenbacher PA, Li S, Pham TD, Pak JE, Chiu W, Kim PS (2021). A single immunization with spike-functionalized ferritin vaccines elicits neutralizing antibody responses against SARS-CoV-2 in mice. ACS Cent Sci.

[138] Geng Q, Tai W, Baxter VK, Shi J, Wan Y, Zhang X, Montgomery SA, Taft-Benz SA, Anderson EJ, Knight AC (2021). Novel virus-like nanoparticle vaccine effectively protects animal model from SARS-CoV-2 infection. PLoS Pathog.

[139] Ma X, Zou F, Yu F, Li R, Yuan Y, Zhang Y, Zhang X, Deng J, Chen T, Song Z (2020). Nanoparticle vaccines based on the receptor binding domain (RBD) and heptad repeat (HR) of SARS-CoV-2 elicit robust protective immune responses. Immunity.

[140] Tabish TA, Hamblin MR (2020). Multivalent nanomedicines to treat COVID-19: A slow train coming. Nano Today.

[141] Lim HX, Lim J, Jazayeri SD, Poppema S, Poh CL (2020). Development of multi-epitope peptide-based vaccines against SARS-CoV-2. Biomed J.

[142] Keech C, Albert G, Cho I, Robertson A, Reed P, Neal S, Plested JS, Zhu M, Cloney-Clark S, Zhou H (2020). Phase 1–2 trial of a SARS-CoV-2 recombinant spike protein nanoparticle vaccine. N Engl J Med.

[143] Ansari MA, Almatroudi A, Alzohairy MA, AlYahya S, Alomary MN, Al-Dossary HA, Alghamdi S (2020). Lipid-based nano delivery of Tat-peptide conjugated drug or vaccine–promising therapeutic strategy for SARS-CoV-2 treatment. Expert Opin Drug Deliv.

[144] Mogheri F, Jokar E, Afshin R, Akbari AA, Dadashpour M, Firouzi-amandi A, Serati-Nouri H, Zarghami N (2021). Co-delivery of metformin and silibinin in dual-drug loaded nanoparticles synergistically improves chemotherapy in human non-small cell lung cancer A549 cells. J Drug Deliv Sci Technol.

[145] Adlravan E, Nejati K, Karimi MA, Mousazadeh H, Abbasi A, Dadashpour M (2021). Potential activity of free and PLGA/PEG nanoencapsulated nasturtium officinale extract in inducing cytotoxicity and apoptosis in human lung carcinoma A549 cells. J Drug Deliv Sci Technol.

[146] Ullah A, Qazi J, Rahman L, Kanaras AG, Khan WS, Hussain I, Rehman A (2020). Nanoparticles-assisted delivery of antiviral-siRNA as inhalable treatment for human respiratory viruses: a candidate approach against SARS-COV-2. Nano Select.

[147] Neufurth M, Wang X, Tolba E, Lieberwirth I, Wang S, Schröder HC, Müller WE (2020). The inorganic polymer, polyphosphate, blocks binding of SARS-CoV-2 spike protein to ACE2 receptor at physiological concentrations. Biochem Pharmacol.

[148] Lee YY, Park HH, Park W, Kim H, Jang JG, Hong KS, Lee J-Y, Seo HS, Na DH, Kim T-H (2020). Long-acting nanoparticulate DNase-1 for effective suppression of SARS-CoV-2-mediated neutrophil activities and cytokine storm. Biomaterials.

[149] Cavalcanti IDL (2020). Pharmaceutical nanotechnology: which products are been designed against COVID-19?. J Nanopart Res.

[150] Rezaee P, Akbari M, Morad R, Koochaki A, Maaz M, Jamshidi Z. First Principle Simulation of Coated Hydroxychloroquine on Ag, Au and Pt Nanoparticle as a Potential Candidate for Treatment of SARS-CoV-2 (COVID-19). arXiv preprint arXiv:200602343 2020. 10.1038/s41598-021-81617-6 PMC7822900 33483539

[151] Lammers T, Sofias AM, van der Meel R, Schiffelers R, Storm G, Tacke F, Koschmieder S, Brümmendorf TH, Kiessling F, Metselaar JM (2020). Dexamethasone nanomedicines for COVID-19. Nat Nanotechnol.

[152] Campos EV, Pereira AE, de Oliveira JL, Carvalho LB, Guilger-Casagrande M, de Lima R, Fraceto LF (2020). How can nanotechnology help to combat COVID-19? Opportunities and urgent need. J Nanobiotechnol.

[153] Mukherjee S, Mazumder P, Joshi M, Joshi C, Dalvi SV, Kumar M (2020). Biomedical application, drug delivery and metabolic pathway of antiviral nanotherapeutics for combating viral pandemic: a review. Environ Res.

[154] Nasrollahzadeh M, Sajjadi M, Soufi GJ, Iravani S, Varma RS (2020). Nanomaterials and nanotechnology-associated innovations against viral infections with a focus on coronaviruses. Nanomaterials.

[155] Hassanzadeh P (2020). Nanotheranostics against COVID-19: From multivalent to immune-targeted materials. J Control Release.

[156] Palmieri V, Papi M (2020). Can graphene take part in the fight against COVID-19?. Nano Today.

[157] Bhavana V, Thakor P, Singh SB, Mehra NK (2020). COVID-19: Pathophysiology, treatment options, nanotechnology approaches, and research agenda to combating the SARS-CoV2 pandemic. Life Sci.

[158] Kalkal A, Allawadhi P, Pradhan R, Khurana A, Bharani KK, Packirisamy G (2021). Allium sativum derived carbon dots as a potential theranostic agent to combat the COVID-19 crisis. Sens Int.

[159] Abo-Zeid Y, Ismail NS, McLean GR, Hamdy NM (2020). A molecular docking study repurposes FDA approved iron oxide nanoparticles to treat and control COVID-19 infection. Eur J Pharm Sci.

[160] Mehranfar A, Izadyar M (2020). Theoretical design of functionalized gold nanoparticles as antiviral agents against severe acute respiratory syndrome coronavirus 2 (SARS-CoV-2). J Phys Chem Lett.

[161] Zhang Q, Honko A, Zhou J, Gong H, Downs SN, Vasquez JH, Fang RH, Gao W, Griffiths A, Zhang L (2020). Cellular nanosponges inhibit SARS-CoV-2 infectivity. Nano Lett.

[162] Cai X, Prominski A, Lin Y, Ankenbruck N, Rosenberg J, Chen M, Shi J, Chang EB, Penaloza-MacMaster P, Tian B (2020). A Neutralizing Antibody-Conjugated Photothermal Nanoparticle Captures and Inactivates SARS-CoV-2. Biorxiv.

[163] Chang S-Y, Huang K-Y, Chao T-L, Kao H-C, Pang Y-H, Lu L, Chiu C-L, Huang H-C (2020). Nanoparticle composite TPNT1 is effective against SARS-CoV-2 and influenza viruses. Gut.

[164] Chauhan G, Madou MJ, Kalra S, Chopra V, Ghosh D, Martinez-Chapa SO (2020). Nanotechnology for COVID-19: therapeutics and vaccine research. ACS Nano.

[165] Lauster D, Glanz M, Bardua M, Ludwig K, Hellmund M, Hoffmann U, Hamann A, Böttcher C, Haag R, Hackenberger CP (2017). Multivalent peptide-nanoparticle conjugates for influenza-virus inhibition. Angew Chem Int Ed.

[166] Ghaffari H, Tavakoli A, Moradi A, Tabarraei A, Bokharaei-Salim F, Zahmatkeshan M, Farahmand M, Javanmard D, Kiani SJ, Esghaei M (2019). Inhibition of H1N1 influenza virus infection by zinc oxide nanoparticles: another emerging application of nanomedicine. J Biomed Sci.

[167] Wang W, Zhou X, Bian Y, Wang S, Chai Q, Guo Z, Wang Z, Zhu P, Peng H, Yan X (2020). Dual-targeting nanoparticle vaccine elicits a therapeutic antibody response against chronic hepatitis B. Nat Nanotechnol.

[168] Palacios R, Patiño EG, de Oliveira PR, Conde MTRP, Batista AP, Zeng G, Xin Q, Kallas EG, Flores J, Ockenhouse CF (2020). Double-Blind, Randomized, Placebo-Controlled Phase III Clinical Trial to Evaluate the Efficacy and Safety of treating Healthcare Professionals with the Adsorbed COVID-19 (Inactivated) Vaccine Manufactured by Sinovac–PROFISCOV: A structured summary of a study protocol for a randomised controlled trial. Trials.

[169] Hoai TT, Yen PT, Dao TTB, Long LH, Anh DX, Minh LH, Anh BQ, Thuong NT (2020). Evaluation of the cytotoxic effect of rutin prenanoemulsion in lung and colon cancer cell lines. J Nanomater.

[170] van Doremalen N, Lambe T, Spencer A, Belij-Rammerstorfer S, Purushotham JN, Port JR, Avanzato VA, Bushmaker T, Flaxman A, Ulaszewska M (2020). ChAdOx1 nCoV-19 vaccine prevents SARS-CoV-2 pneumonia in rhesus macaques. Nature.

[171] Ramasamy MN, Minassian AM, Ewer KJ, Flaxman AL, Folegatti PM, Owens DR, Voysey M, Aley PK, Angus B, Babbage G (2020). Safety and immunogenicity of ChAdOx1 nCoV-19 vaccine administered in a prime-boost regimen in young and old adults (COV002): a single-blind, randomised, controlled, phase 2/3 trial. Lancet.

[172] Mercado NB, Zahn R, Wegmann F, Loos C, Chandrashekar A, Yu J, Liu J, Peter L, McMahan K, Tostanoski LH (2020). Single-shot Ad26 vaccine protects against SARS-CoV-2 in rhesus macaques. Nature.

[173] Poland GA, Ovsyannikova IG, Kennedy RB (2020). SARS-CoV-2 immunity: review and applications to phase 3 vaccine candidates. Lancet.

[174] Raja AT, Alshamsan A, Al-Jedai A (2020). Current COVID-19 vaccine candidates: implications in the Saudi population. Saudi Pharma J.

[175] Jackson LA, Anderson EJ, Rouphael NG, Roberts PC, Makhene M, Coler RN, McCullough MP, Chappell JD, Denison MR, Stevens LJ (2020). An mRNA vaccine against SARS-CoV-2—preliminary report. N Engl J Med.

[176] Mulligan MJ, Lyke KE, Kitchin N, Absalon J, Gurtman A, Lockhart S, Neuzil K, Raabe V, Bailey R, Swanson KA (2020). Phase I/II study of COVID-19 RNA vaccine BNT162b1 in adults. Nature.

[177] Rawat K, Kumari P, Saha L (2021). COVID-19 vaccine: A recent update in pipeline vaccines, their design and development strategies. Eur J Pharmacol.

[178] Sahin U, Muik A, Derhovanessian E, Vogler I, Kranz LM, Vormehr M, Baum A, Pascal K, Quandt J, Maurus D (2020). COVID-19 vaccine BNT162b1 elicits human antibody and TH 1 T cell responses. Nature.

[179] Richner JM, Himansu S, Dowd KA, Butler SL, Salazar V, Fox JM, Julander JG, Tang WW, Shresta S, Pierson TC (2017). Modified mRNA vaccines protect against Zika virus infection. Cell.

[180] Sekimukai H, Iwata-Yoshikawa N, Fukushi S, Tani H, Kataoka M, Suzuki T, Hasegawa H, Niikura K, Arai K, Nagata N (2020). Gold nanoparticle-adjuvanted S protein induces a strong antigen-specific IgG response against severe acute respiratory syndrome-related coronavirus infection, but fails to induce protective antibodies and limit eosinophilic infiltration in lungs. Microbiol Immunol.

[181] Allawadhi P, Khurana A, Allwadhi S, Joshi K, Packirisamy G, Bharani KK (2020). Nanoceria as a possible agent for the management of COVID-19. Nano Today.

[182] Hassani N, Jafari-Gharabaghlou D, Dadashpour M, Zarghami N. The effect of dual bioactive compounds artemisinin and metformin co-loaded in PLGA-PEG nano-particles on breast cancer cell lines: potential apoptotic and anti-proliferative action. Appl Biochem Biotechnol. 2022:1–6. 10.1007/s12010-022-04000-9 35674922

